# A reduction-sensitive lipophilic dihydroartemisinin prodrug in a self-microemulsifying drug delivery system for treating breast cancer lung metastasis via intestinal lymphatic transport

**DOI:** 10.1016/j.ijpx.2026.100556

**Published:** 2026-05-03

**Authors:** Bin Zheng, Cuiping He, Fengye Zhao, Ran Li, Ziyi Zhang, Xiaojie Chen, Minfei Shi, Beibei He, Rongrong Wang, Guolian Ren, Shuqiu Zhang, Shuang Yang

**Affiliations:** a Medicinal Basic Research Innovation Center of Chronic Kidney Disease, Ministry of Education, School of Pharmacy, Shanxi Medical University, Taiyuan 030001, China; b Shanxi Provincial Key Laboratory of Drug Synthesis and Novel Pharmaceutical Preparation Technology, School of Pharmacy, Shanxi Medical University, Taiyuan 030001, China; c School of Basic Medical Sciences, Shanxi Medical University, Taiyuan 030001, China

**Keywords:** Breast cancer metastasis, Intestinal lymphatic transport, Self-microemulsifying drug delivery system, Lipophilic prodrug, Reduction-sensitive, Dihydroartemisinin

## Abstract

The eradication of cancers within the lymphatic system is a key treatment goal for cancer metastasis and an important determinant of patient prognosis. Therefore, efficient delivery of chemotherapy drugs to the lymphatic system with minimal side effects holds potential for improved treatment options in aggressive breast cancer. In contrast to the invasive administration routes, oral nanocarriers, particularly self-microemulsifying drug delivery system (SME) has attracted increasing attention to treating lymphatic disorders by exploiting intestinal lymphatic transport. In this study, an optimized SME formulation loaded with a reduction-sensitive lipophilic dihydroartemisinin (DHA) prodrug (DSC) was developed for oral delivery and the treatment of breast cancer metastasis by harnessing intestinal lymphatic transport. Compared with parent DHA, DSC exhibited higher affinity with the oil phase of SME, improving the molar drug loading (1.83-fold) and sustained-release behavior of the SME formulation. DSC also exhibited reduction-triggered release of DHA under high reducing condition, which may reduce unnecessary systemic exposure while improving antitumor efficacy. In vitro cell studies revealed that DSC-SME was internalized into intestinal epithelial cells primarily via caveolae/lipid raft- and clathrin-mediated endocytosis, rather than macropinocytosis. Following internalization, it was subsequently trafficked across cells via the chylomicron pathway. After oral administration, DSC-SME enhanced intestinal retention and promoted drug accumulation in mesenteric lymph nodes. Its oral bioavailability was 8.91- and 2.14-fold higher than that of free DHA and DHA-SME, respectively. Both in vitro and in vivo studies indicated that DSC-SME exhibited favorable antitumor efficacy against murine orthotopic 4 T1 breast tumors and lung metastases, with no significant gastrointestinal or systemic toxicity observed under the current experimental conditions. The proposed mechanism of action involved glutathione depletion, apoptosis induction, proliferation inhibition, and amelioration of the immunosuppressive tumor microenvironment. Collectively, these findings suggest that integrating a reduction-responsive lipophilic prodrug with a convenient SME formulation may offer a promising oral platform for inhibiting cancer metastasis, warranting further preclinical evaluation.

## Graphical abstract

**Figure:**
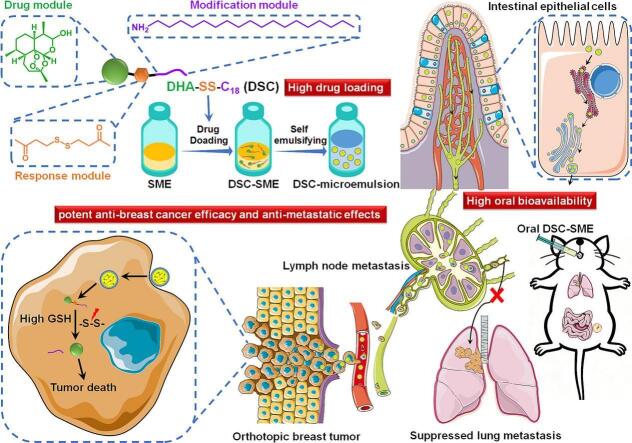

## 1 Introduction

Breast cancer has overtaken lung cancer as the most common type of cancer worldwide, seriously threating to women life and health (Thakur et al., 2024). In the year 2025, it is estimated that 319,750 people (including 316,950 women) will be diagnosed with breast cancer and 226,650 people (including 115,970 women) with lung & bronchus cancer in the USA (Siegel et al., 2025). Most deaths caused by aggressive breast cancer are not due to the primary tumor itself, but are the result of cancer metastasis to lymph nodes and systemic sites in the body (Park et al., 2022). A growing body of evidence highlights the critical role of the lymphatic system in early-stage metastasis. This process begins when malignant epithelial cells breach the basal lamina of the breast duct, a transition that marks the shift from in situ disease to invasive carcinoma. Subsequently, these tumor cells invade the lymphatic vessels, travel to regional lymph nodes, and may ultimately colonize distant organs (Mahjabeen et al., 2025).

Recently, targeted therapy and immunotherapy have shown efficacy against breast cancer, but the core challenges that limit their effectiveness remain the multi-level mechanisms of drug resistance, the immunosuppressive tumor microenvironment, and the absence of reliable predictive biomarkers (Ye et al., 2023). Given the limitations of emerging therapies, chemotherapy, whether as a standalone treatment or in combination with surgery and radiotherapy, is still the preferred clinical strategy for the prevention and treatment of cancer metastasis (D'Alterio et al., 2020). However, conventional small-molecule chemotherapy drugs inefficiently access the lymphatic system, making the effective eradication of lymphatic metastases difficult without causing toxicity to other normal tissues (Wang et al., 2019). Thus, novel drug formulations or nano drug delivery systems (NDDS) provide a potential platform to promote delivery of chemotherapeutics to lymphatic system, which may improve long-term outcomes in cancer treatment.

Widely investigated in cancer therapy, NDDS have demonstrated a range of potential advantages for systemic chemotherapy, many of which are also applicable to the treatment of tumor lymphatic metastases (Ryan et al., 2014). A key advantage of NDDS is their propensity to drain into the lymphatic system (McCright et al., 2022). This occurs not only after interstitial administration (intramuscular, intradermal, or subcutaneous) but also, in certain instances, following intravenous injection, where they can traverse systemic capillary beds to reach lymphatics (Vishwakarma et al., 2019). Lymphatic uptake has also been reported for certain NDDS following oral administration (Zhang et al., 2021). In contrast to the invasive routes mentioned above, oral administration offers distinct advantages such as ease of use, favorable safety, and dosing flexibility. These benefits collectively enhance patient compliance and quality of life, which is particularly valuable in long-term breast cancer management (Afrin et al., 2023). In the intestine, the chylomicrons in enterocytes and the microfold cells (M cells) in Peyer's patches have been established as major portals for intestinal lymphatic transport (Elz et al., 2022; Zheng et al., 2024). Efficient delivery of therapeutic agents to the intestinal lymphatic system offers two key advantages: (i) it enhances oral drug bioavailability by circumventing first-pass hepatic metabolism; and (ii) it enables direct treatment of lymphatic system-related pathologies, including lymphoma, solid tumor metastases, HIV infection, diabetes, lymphedema, schizophrenia, and tuberculosis. This strategy simultaneously improves therapeutic efficacy while minimizing systemic side effects (Bachhav et al., 2017; Cao et al., 2021; Feeney et al., 2020; Miao et al., 2021; Qin et al., 2021). Intestinal lymphatic transport can be achieved via formulation strategies, such as incorporating lipid digestion enhancers (e.g. bile salts), designing high lipophilicity drugs (logP >5) and employing lipid-based nanocarriers with high long-chain triglyceride solubility polymer (> 50 mg/g). Regarding the properties of lipid-based nanocarriers, key parameters include particle size (preferably <100 nm), molecular weight (10–16 kDa), and surface charge (negatively charged > positively charged > neutral) (Vishwakarma et al., 2019).

In recent years, various lipid-based nanocarriers including liposome, solid lipid nanoparticle, nanostructured lipid carrier, nano/micro-emulsion, and self-microemulsifying drug delivery system (SME) have been extensively investigated for their ability to promote drugs delivery into the intestinal lymphatic system (Gao et al., 2021; Koli et al., 2021; Meshram and Ranpise, 2024; Shrivastava and Kaur, 2023; Ye et al., 2021; Zheng et al., 2024). SME, is a nanocarrier composed of oil phase, surfactant, and co-surfactant, primarily addressing the absorption challenges of hydrophobic or hydrolysis-prone drugs (Hwang et al., 2026). Upon oral administration, it spontaneously forms an oil-in-water microemulsion in situ with a droplet size typically below 100 nm by gastrointestinal fluid (Protopapa et al., 2026). This significantly enhances drug oral bioavailability by mechanisms such as increasing solubility, protecting against gastrointestinal degradation, promoting permeability, and enhancing intestinal lymphatic transport (Paliwal et al., 2024). Furthermore, SME exhibits high stability, enables controlled drug release, and offers a relatively low production cost (Salawi, 2022). The efficacy of SME in treating lymphatic disorders is evidenced by several marketed products, including the antiretroviral Norvir® (ritonavir), the immunosuppressant Neoral® (cyclosporine), and the antineoplastic agent Targretin® (bexarotene) (Rani et al., 2019).

Dihydroartemisinin (DHA), the active metabolite of artemisinin and its derivatives, is a clinically established antimalarial agent, which has recently emerged as a promising candidate for drug repurposing in cancer therapy (Wong et al., 2022; Wu et al., 2026). Researches indicate that DHA exerts its anticancer activity primarily through ferrous iron (Fe^2+^)-triggered cleavage of its endoperoxide bridge, leading to a burst of reactive oxygen species (ROS) that induces mitochondrial dysfunction, DNA damage, and multiple programmed cell death pathways including apoptosis and ferroptosis. Additionally, DHA directly targets key proteins such as ANXA2 and EGFR to inhibit the PI3K/AKT and NF-κB pathways, thereby suppressing tumor angiogenesis, invasion, and lymphatic metastasis while sparing normal cells due to their lower intracellular iron levels (Dai et al., 2021; Wen et al., 2024; Wu et al., 2025a). As a repurposed drug with proven safety and low cost, DHA offers a time-efficient and cost-effective option to accelerate the development of cancer therapy, complementing existing therapeutic strategies.

Given the low water solubility, poor stability, and extensive first pass metabolism exhibited by DHA, many studies have focused on developing DHA-loaded SME formulations to enhance the oral bioavailability of DHA (Srivastava et al., 2022; Wang et al., 2022). In these studies, two kinds of DHA-SME were constructed using different excipients, and the formulations were optimized via ternary phase diagrams. Although corresponding in vitro studies were conducted, there is a lack of research on pharmacokinetics and lymphatic transport. More importantly, a major formulation challenge for DHA-loaded SME is the poor compatibility of DHA with conventional oil phases. This may lead to low encapsulation efficiency, inadequate colloidal stability, and consequent drug leakage in the gastrointestinal tract.

The prodrug approach has been extensively utilized to overcome inherent flaws of active compounds, such as poor solubility, instability, low absorption, unfavorable distribution, and toxicity (Chien et al., 2023; Markovic et al., 2019). Therefore, designing lipophilic DHA prodrugs represents a rational strategy to enhance their affinity for the oil phase of SME. Meanwhile, to maximize the antitumor efficacy and minimize systemic toxicity of DHA prodrugs, it is crucial that the linkers in prodrugs are selectively cleaved at the tumor site to release the parent drug DHA (Bargakshatriya and Pramanik, 2023). The disulfide bond is a reduction-sensitive linkage that is cleaved by glutathione (GSH), whose concentration is highly upregulated in tumor microenvironment and tumor cells, enabling tumor-specific drug release (Sun et al., 2024; Zhang et al., 2024).

**Scheme 1:**
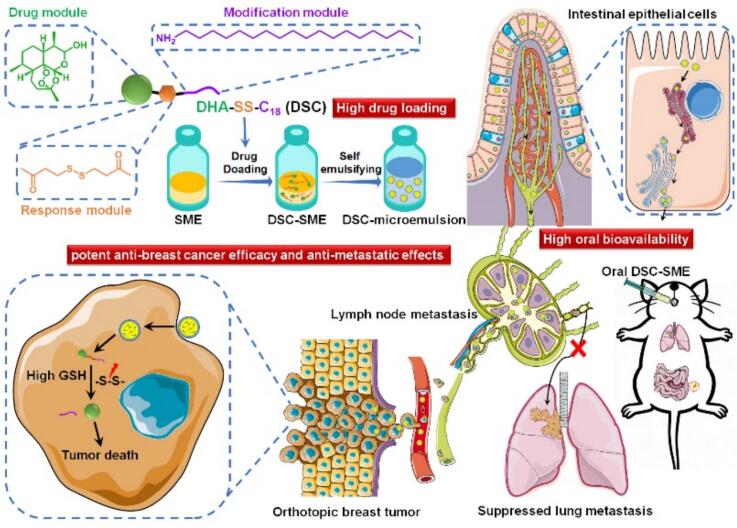
Schematic illustration of the construction process, intestinal lymphatic transport, and anti-breast cancer metastatic action of DSC-SME.

While DHA prodrug design (for improved solubility) and SME formulation (for enhanced lymphatic transport) have each been reported separately, their synergistic integration for DHA-based lymphatic-targeted therapy of breast cancer metastasis remains unexplored. Prior approaches either generated lipophilic DHA prodrugs without a lymph-targeted carrier, or used SMEs to deliver unmodified DHA with poor drug loading. Moreover, the compatibility between DHA and the carrier has rarely been addressed in existing DHA delivery systems. To fill these gaps, this study designed a reduction-sensitive lipophilic DHA prodrug-loaded SME system, with the aim of enhancing lymphatic targeting and improving DHA's antitumor efficacy against breast cancer lymphatic metastasis. As shown in Scheme 1, the DHA-SS-C18 prodrug named as DSC is designed and synthesized by conjugating DHA with octadecylamine via a disulfide bond linker. An oral DSC loaded SME (DSC-SME) is developed for targeted delivery of DSC to the lymphatic system via the chylomicron pathway. This lymphatic transport route enables DSC-SME bypassing the hepatic entry and directly entering the systemic circulation, thereby avoiding first-pass metabolism and significantly enhancing the oral bioavailability of DHA. Moreover, by achieving high lymphatic distribution, DSC-SME exerts not only direct antitumor effects on murine orthotopic 4 T1 breast tumor, but also demonstrates favorable anti-metastatic activity against lung metastases.

## 2 Materials and methods

### 2.1 Materials

Dihydroartemisinin (DHA) was purchased from Huali Wulingshan Pharmaceutical Co., Ltd. (Chongqing, China). Octanedioic acid, 3,3′-dithiodipropionic acid, 4-dimethylaminopyridine, N-hydroxysuccinimide, N,N′-dicyclohexylcarbodiimide, octadecylamine, cycloheximide (CHX), DiR, ammonium acetate, triethanolamine, n-butanol, and cremophor EL were purchased from Aladdin (Shanghai, China). Medium chain triglycerides (MCT) was purchased from OriLeaf (Shanghai, China). Methyl-β-cyclodextrin (M-β-CD), chlorpromazine hydrochloride (CPZ), coumarin-6, and pancreatin were purchased from Macklin (Shanghai, China). Fetal bovine serum (FBS) was purchased from Cellmax (Beijing, China). Phosphate buffer saline (PBS), 4% paraformaldehyde, lomitapide, penicillin/streptomycin solution, Bouin's dye solution and AnnexinV-FITC/PI cell apoptosis detection kit were obtained from Meilun Bio Company (Dalian, China). DAPI, Hanks' balanced salt solution (with Ca^2+^ & Mg^2+^, HBSS) and Dulbecco's modified Eagle's medium (DMEM) were acquired from Boster Bio Company (Wuhan, China). ER-Tracker, Lyso-Tracker, Golgi-Tracker, and ROS detection kit were acquired from Beyotime Biotechnology (Shanghai, China). Bafilomycin A1, ethylisopropylamiloride (EIPA), brefeldin A, monensin sodium salt, and dynasore were acquired from MedChemExpress (New Jersey, USA). Alanine aminotransferase assay kit, aspartate aminotransferase assay kit, alkaline phosphatase assay kit, urea assay kit, and creatinine assay kit were acquired from Nanjing Jiancheng Bioengineering Institute (Nanjing, China). TNF-α ELISA Kit and VEGF ELISA Kit were acquired from Bioswamp (Wuhan, China). IL-6 ELISA Kit was acquired from Meimian (Nanjing, China). All other analytical reagents were commercially obtained in analytical grade or better.

### 2.2 Cells

Caco-2 human colon adenocarcinoma, 4 T1 murine breast cancer, MCF-7 human breast cancer, and HepG2 human hepatoma cells were purchased from American Type Culture Collection (ATCC). Caco-2 cells were grown in complete high-glucose DMEM containing 20% (*v*/*v*) FBS, 1% (*v*/*v*) nonessential amino acids (NEAA), 1% (*v*/*v*) l-glutamine, and 1% (*v*/*v*) penicillin and streptomycin (P/S). 4 T1, MCF-7 and HepG2 cells were grown in complete high-glucose DMEM containing 10% (*v*/*v*) FBS and 1% (*v*/*v*) P/S. The cells were cultured at 37 °C with 90% relative humidity and 5% CO_2_.

### 2.3 Animals

Female BALB/c mice (18 ± 2 g), female ICR mice (29 ± 1 g) and male SD rats (220 ± 20 g) were provided by the Experimental Animal Center of Shanxi Medical University (Taiyuan, China). All mice and rats were housed at a temperature of 25 ± 2 °C under a 12 h light/dark cycle with free access to water and food for 1 week before the experiment. The experimental protocol was approved by the Institutional Animal Care and Use Committee of Shanxi Medical University (No. SYDL2024013) and performed under the Guidelines for Ethical Review of Laboratory Animal Welfare of China (GB/T 35892–2018). Before the experiments, the mice and rats were fasted for over 12 h and had free access to water.

### 2.4 Assay analysis

A UV–Vis spectrophotometric method was used for the determination of DHA, DCC and DSC content in drug loading capacity, encapsulation efficiency (EE), and in vitro release studies. In brief, the DHA, DCC and DSC samples were dissolved in an appropriate amount of ethanol, and then mixed with 4 times the volume of 10% NaOH. The mixed sample was incubated in a water bath at 60 °C for 2 h, and then cooled to room temperature (Zheng et al., 2024). The absorbance was measured at 238 nm by a UV–Vis spectrophotometer (UV-1200, NAPADA, Shanghai, China).

The content of DHA, DCC and DSC content in purity identification and in vitro lipolysis studies were determined using high-performance liquid chromatography (HPLC) with post-column derivatization method (Wang et al., 2025). For DHA analysis, the HPLC system was equipped with an Agilent®C18 column (150 × 4.6 mm, 5.0 μm). The mobile phase consisted of methanol/acetonitrile/ammonium acetate buffer (20:50:30, *v*/*v*/v) and was delivered at a flow rate of 0.8 mL/min. The retention time of DHA was 5.2 min. For the lipophilic DHA prodrugs (DCC and DSC), a Kinetex® C8 column (50 × 2.1 mm, 2.6 μm) was used, with the mobile phase modified to methanol/acetonitrile/ammonium acetate buffer (30:55:15, *v*/*v*/*v*) at a flow rate of 0.4 mL/min. The detection wavelength for DHA, DCC, and DSC was 289 nm. The injection volume was 10 μL, and the column temperature was maintained at 40 °C. The retention times of DCC and DSC were 5.1 and 5.1 min, respectively.

To avoid interference from DHA on its prodrugs (DCC and DSC) in the process of assay analysis, the concentration of DHA, DCC and DSC in samples obtained from the reductive responsive release and pharmacokinetic studies were measured with a validated HPLC–MS/MS method using artemisinin (ART) as an internal standard after extraction with ether (Chai et al., 2021). The chromatographic separation of prepared samples was performed using an HPLC system (Agilent 1200, Agilent Technologies Inc., California, USA) equipped with a Kinetex®C8 column (50 × 2.1 mm, 2.6 μm) and a C8 precolumn (4.0 × 3.0 mm, 5.0 μm). The mobile phase was composed of acetonitrile and 10 mM ammonium acetate (90:10, *v*/*v*) and was run at 0.3 mL/min. The injection volume was 10 μL, and the column temperature was maintained at 30 °C. The retention times of ART, DHA, DCC, and DSC were 0.6, 0.6, 2.7, and 2.6 min, respectively. The mass spectrometer (API 3000, AB SCIEX LLC., California, USA) was equipped with electrospray ionization (ESI) and operated in positive mode. Multiple-reaction monitoring (MRM) transitions for DHA, DCC, DSC, and ART (internal standard) were *m*/*z* 302.5 → 163.3, m/z 709.5 → 408.6, m/z 745.4 → 356.5, and m/z 300.3 → 209.2, respectively. The instrument was operated with an ion spray voltage of 5500 V, curtain gas pressure of 10 psi, nebulizer gas pressure of 65 psi, and heater gas pressure of 30 psi. The heater gas temperature was set at 300 °C. The declustering potentials used for the analysis of DHA, DCC, DSC, and ART were 24.0 V, 51.0 V, 73.9 V, and 25.0 V, respectively. The collision energies for DHA, DCC, DSC, and ART were 21.0 V, 40.1 V, 39.6 V, and 20.0 V, respectively.

### 2.5 Synthesis of DHA prodrugs

The DHA prodrugs were denoted as DHA-CC-C18 (DCC) and DHA-SS-C18 (DSC). Briefly, octanedioic acid or 3,3′- dithiodipropionic acid (0.95 mmol) was added to 3 mL of acetic anhydride, and reacted at 30 °C under magnetic stirring for 3.5 h. Then unreacted acetic anhydride was removed by reducing the pressure with adding methylbenzene. The obtained solid product was dissolved in 15 mL of dichloromethane, and then DHA (0.32 mmol) and 4-dimethylaminopyridine (0.03 mmol) were added dropwise, and reacted at 35 °C under magnetic stirring. After 36 h, the crude product was separated and purified by silica gel column chromatography (eluent: cyclohexane/ethyl acetate, gradient from 5:1 to 3:1). The obtained product (1.05 mmol), N-hydroxysuccinimide (1.25 mmol) and N,N′-dicyclohexylcarbodiimide (1.25 mmol) were added to 10 mL of dichloromethane in sequence, and reacted at 0 °C under magnetic stirring for 4 h. Then the reaction was carried out at room temperature for another 20 h. The reaction mixture was filtered to remove solid precipitates, and then octadecylamine (C18-NH_2_, 1.25 mmol) was added to the filtrate. After 48 h of reaction at room temperature, the final product DCC or DSC was separated and purified by silica gel column chromatography (eluent: n-hexane/ethyl acetate, gradient from 3:1 to 1:1). The structures of DCC and DSC were confirmed by high resolution mass spectrometry (HRMS, Thermo Scientific LTQ Orbitrap XL) and nuclear magnetic resonance spectroscopy (^1^H NMR, AVANCE NEO 400 MHz, Bruker, Swizerland). The purities of DCC and DSC were determined via the HPLC with post-column derivatization method described in the “Assay analysis” section and were calculated by peak area normalization.

### 2.6 Solubility study

The solubilites of DHA, DCC, and DSC in various oils (ethyl oleate, castor oil, olive oil, MCT, and IPM) and cosurfactants (triethanolamine, transcutol HP, PEG400, 1,2-propanediol, and n-butanol) were determined using the shake flask method (Wang et al., 2023). Excess amount of DHA, DCC, or DSC were added to 1.0 g of each excipient in the 5 mL centrifugal tube, respectively. The mixtures were vortexed for 2 min and shaken at 150 rpm for 48 h at 37 ± 0.5 °C in a gas bath constant temperature oscillator (SHA-BA, Jintan Ronghua Instrument Manufacture Co., Ltd., Jiangsu, China). The equilibrated mixtures were centrifuged at 7000 rpm for 20 min to remove the undissolved drugs. The content of DHA, DCC, and DSC in the supernatant was measured by the UV–Vis spectrophotometric method described in the “Assay analysis” section.

### 2.7 Compatibility study

#### 2.7.1 Compatibility of oil and surfactant

The total mass was kept at 100 mg based on the result of solubility study, and the selected oils were mixed with selected surfactants (Tween-20, Tween-80, OP-10, cremophor EL, RH40, and labrasol) in varying mass ratios (1:9, 2:8, 3:7, 4:6, and 5:5) (Wang et al., 2023). The mixtures were vortexed for 2 min and emulsified with 10 mL of distilled water at 37 ± 0.5 °C under magnetic stirring. The state of the mixture was visually judged for the rate of emulsification and final appearance. The mixture was graded as a Grade “I” microemulsion if formed (< 1 min) with a clear or micro-blue transparent appearance or a Grade “II” microemulsion if formed (< 2 min) with a slightly less clear appearance.

#### 2.7.2 Compatibility of surfactant and cosurfactant

Based on the result of solubility study, the selected cosurfactants and surfactants were mixed in the mass ratio of 1:1. The mixtures were vortexed for 3 min, centrifuged at 7000 rpm for 5 min, and then observed whether there was stratification. If there was no stratification, it indicated that the surfactant is compatible with the cosurfactant, represented by the letter “P”. On the contrary, it was incompatible and was represented by the letter ‘N'.

### 2.8 Formulation development

#### 2.8.1 Pseudo-ternary phase diagrams

The pseudo-ternary phase diagram was constructed to further screen the optimal components to obtain self-microemulsification (without DHA or prodrugs). Self-emulsifying regions were identified within this diagram by visually observation with varying the ratio of each selected excipient. Briefly, a series of SME formulations were prepared employing MCT as oil, cremophor EL/RH40 as surfactants, and transcutol HP/n-butanol as cosurfactants (based on the results of solubility and compatibility studies). The mass ratios of surfactant to cosurfactant (Km) were 9:1, 8:2, 7:3, 6:4, 5:5, 4:6, 3:7, 2:8, and 1:9, while the varying mass ratios of oil to the surfactant/cosurfactant mixture (K) were blended at 0.5:9.5, 1:9, 1.5:8.5, 2:8, 2.5:7.5, 3:7, 3.5:6.5, 4:6, 4.5:5.5, and 5:5. Then the above mixtures (100 mg) was added into 10 mL of distilled water, and stirred magnetically at 37 ± 0.5 °C. The self-emulsification process was also visually evaluated and the composition of the mixtures labeled Grade “I” and “II” were noted, and then the pseudo-ternary phase diagrams were plotted using Origin software.

#### 2.8.2 Effect of the K value on droplet size

To further study the effect of the K value on emulsion droplet size, a series of SME formulations were prepared with the Km value fixed at 7:3. The K value was increased from 1:9 to 5:5. Each formulation (100 mg) was dispersed into 10 mL of distilled water (37 °C) under gentle stirring and the droplet size was determined by a dynamic light scattering analyzer (Zetasizer Nano ZS, Malvern, UK).

### 2.9 Preparation and characterization of drug-loaded SME

The optimal blank self-microemulsifying drug delivery system (SME) was prepared by mixing 15% MCT (oil), 59.5% Cremophor EL (surfactant), and 25.5% n-butanol (cosurfactant) under stirring (250 rpm) at 37 °C for 10 min. To explore the equilibrium solubility in SME, excess amounts of DHA, DCC, and DSC were added into the blank SME, and then mixed in a gas bath constant temperature oscillator for 24 h at 37 ± 0.5 °C. The equilibrated mixtures were centrifuged at 7000 rpm for 20 min to remove the undissolved drugs. The content of DHA, DCC, and DSC in the supernatant was measured by the UV–Vis spectrophotometric method described above. The optimal drug loading capacity (DL) of drug-loaded SME formulations (DHA-SME, DCC-SME, and DSC-SME) was determined by observing whether there were drug precipitation and the ability to regenerate microemulsion after 4 °C of storage, and measuring the EE of drug-loaded microemulsions. The drug-loaded microemulsion was formed by adding 100 mg of drug-loaded SME into 10 mL of distilled water (37 °C) under gentle stirring. The low-speed centrifugation method (7,000 rpm, 15 min) was used to separate drug-loaded microemulsion and non-encapsulated drug. The EE of drug-loaded microemulsion was calculated using Eq. (1):

(1) $$\mathrm{EE}\;\left(\%\right)=W_{E}/W_{T}\times 100$$

where W_T_ was the weight of drug added in the preparation process, and W_E_ was the weight of drug encapsulated in microemulsion, which was derived from the supernatant of drug-loaded microemulsion.

The droplet size, polydispersity indexes (PDI), and zeta potential of drug-loaded microemulsions were measured by a dynamic light scattering analyzer. The morphological features of drug-loaded microemulsions were investigated by transmission electron microscopy (TEM, JEM-1200EX, JEOL, Japan). The samples were pretreated with negative staining using uranyl acetate on a copper grid. Robustness of DHA-SME, DCC-SME, and DSC-SME to dilution was determined by measuring the droplet size after diluting with hydrochloric acid (pH 1.2) to different extent (1:5, 1:10, 1:20, 1:50, 1:100, and 1:500, *v*/*v*).

### 2.10 Medium and storage stability study

To test the stability of DHA-SME, DCC-SME, and DSC-SME in the gastrointestinal tract in vitro, 20 mg drug-loaded SME were dispersed in a certain volume of simulated gastric fluid (SGF, pH 1.2), simulated intestinal fluid (SIF, pH 6.8), or PBS (pH 7.4). These drug-loaded microemulsions were incubated at 37 ± 0.5 °C and shaken at 150 rpm. At predetermined time points (0, 1, 2, and 4 h), the droplet size of drug-loaded microemulsions in SGF was measured as described above. For the stability of drug-loaded microemulsions in SIF and PBS, the droplet size was measured at 0, 8, 16, and 24 h. The storage stability of DHA-SME, DCC-SME, and DSC-SME was evaluated by measuring the changes in droplet size of drug-loaded microemulsions for up to 90 days at 4 °C and room temperature.

### 2.11 In vitro release assay

An in vitro release assay was carried out by a dynamic dialysis method in a gas bath constant temperature oscillator to evaluate the release characteristics of DHA, DCC, and DSC from the drug-loaded microemulsions. Briefly, DHA-SME, DCC-SME, or DSC-SME was dispersed into distilled water to form drug-loaded microemulsions, and the equivalent DHA concentrations were 0.6 mg/mL. Then, 2 mL of drug-loaded microemulsions or DHA suspension (DHA-SUS, DHA highly dispersed in 0.5% *w*/*v* carboxymethyl cellulose sodium aqueous solution) were placed in a dialysis bag (MW cut-off 8–14 kDa). To mimic the release situation of drug-loaded microemulsions in the gastrointestinal environment, the dialysis bag was first incubated in 20 ml of SGF for 2 h, and then incubated in 20 ml of SIF for another 22 h at 37 ± 0.5 °C and shaken at 150 rpm. An aliquot of 0.5 ml release medium was withdrawn at the fixed time points (0.5, 1, 2, 3, 4, 6, 8, 12, and 24 h), and an equal volume of fresh release medium was replenished. The concentrations of DHA, DCC, and DSC in the release medium were measured by the UV–Vis spectrophotometric method described in the “Assay analysis” section.

To investigate the DHA release behavior under the reductive environment, prodrug microemulsions were incubated in release medium (PBS, pH 7.4) with 10 mmol/L dithiothreitol (DTT) or GSH at 37 ± 0.5 °C. At the fixed time points, the released DHA amount from the prodrug-loaded microemulsions was measured by the HPLC-MS/MS method described in the “Assay analysis” section. All the experiments were performed in triplicate.

### 2.12 In vitro lipolysis study

The digestion medium was prepared by mixing digestion buffer (50 mM trihydroxymethyl aminomethane, 150 mM NaCl, 5 mM CaCl_2_, pH 7.5) with 1.25 mM egg yolk lecithin and 5 mM sodium taurodeoxycholic acid. Pancreatin extract was freshly prepared in digestion medium (200 mg·mL^−1^ pancreatin) with 30 min of stirring at 4 °C and then centrifuged at 6000 rpm for 30 min (Cui et al., 2019). The supernatant was stored at a temperature of −20 °C until use.

Drug-loaded SME (200 mg) or DHA-SUS (at an amount equivalent to that in DHA-SME) was dispersed in 18 mL of digestion medium, and then mixed with 2 mL of pancreatin extract. The mixture was shaken at 37 ± 0.5 °C. At the fixed time points (5, 15, 30, and 60 min), 1 ml of mixture was withdrawn and 10 μL of 4-bromophenylboronic acid (0.5 M) was added to terminate the lipolysis process. The mixture was centrifuged at 12000 rpm for 30 min under 4 °C, and then 100 μL of middle aqueous layer liquid was taken out and mixed with 200 μL of methanol. The supernatant was collected after centrifuging at 10000 rpm for 10 min. The concentrations of DHA, DCC, and DSC were measured by the HPLC method described in the “Assay analysis” section. All the experiments were performed in triplicate.

### 2.13 Cellular uptake study

The cellular uptake of SME was quantitatively and qualitatively examined by flow cytometry (FCM) and high content cell imaging analysis system (HCS), respectively. The fluorescence-labeled SME was prepared by using coumarin-6 (Cou-6). For FCM, Caco-2 cells were seeded at a density of 1.0 × 10^5^ cells/well into 24-well plates. After 36 h of culture, the cells were incubated with Cou-6 or Cou-6-labeled SME (C6-SME) (Cou-6 equivalent concentration: 100 ng/mL) at 37 °C for 0.5, 1, 2, and 4 h. Cells treated with only high-glucose DMEM were used as a negative control. At the end of the incubation period, the cells were washed thrice with cold PBS (pH 7.4), trypsinized, collected, and fixed with 4% paraformaldehyde. The fluorescence intensity in the cells was analyzed by FCM (FACSCelesta, BD Biosciences, San Jose, CA, USA).

For HCS, Caco-2 cells were incubated with Cou-6 or C6-SME (Cou-6 equivalent concentration: 100 ng/mL) at 37 °C for 0.5, 1, 2, and 4 h. Subsequently, the cells were washed thrice with cold PBS (pH 7.4) and fixed with 4% paraformaldehyde. The cell nuclei were stained with DAPI. The cells were washed thrice with PBS (pH 7.4) and observed by HCS (ImageXpress Pico, Molecular Devices, Silicon Valley, CA, USA). All the experiments were performed in triplicate.

### 2.14 Endocytosis pathways in Caco-2 cells

To explore the endocytosis pathway of SME, Caco-2 cells were preincubated with different endocytosis inhibitors, including CPZ (30 μM), MβCD (10 mM), dynasore (80 μM) and EIPA (100 μM) at 37 °C for 1 h. Subsequently, the cells were incubated with C6-SME for another 2 h. During the incubation period of C6-SME, the concentrations of endocytosis inhibitors were maintained constant. After co-incubation, the cells were washed thrice with cold PBS (pH 7.4), trypsinized, collected, and fixed with 4% paraformaldehyde. The fluorescence intensity in the cells was analyzed by FCM. All the experiments were performed in triplicate.

### 2.15 Transepithelial transport study using the Caco-2 cell monolayers

To construct the Caco-2 cell monolayers, Caco-2 cells were seeded at a density of 1.0 × 10^5^ cells/well on the 12-well transwell plate (membrane material: PET; pore size: 0.4 μm; growth area: 1.12 cm^2^) and cultured for 21 days. The culture medium was replaced every 2 days in the first week and every day since the second week. During 21 days of culture, the transepithelial electrical resistance (TEER) value was measured using a cell resistance meter (RE1600, Kingtech, Beijing, China) to monitor the integrity of cell monolayers. The content of alkaline phosphatase (AKP) in the apical chamber and basolateral chamber was detected to evaluated the cell polarity using an alkaline phosphatase activity detection kit according to the manufacturer's protocol. The Caco-2 cell monolayers could be used for the subsequent transcellular transport study when the TEER value was over 400 Ω·cm^2^. Before the experiments, the medium in the apical chamber and basolateral chamber were replaced with pre-warmed HBSS.

After incubation at 37 °C for 30 min, 0.5 mL of Cou-6 or C6-SME (100 ng/mL Cou-6) were added to the apical chamber. At predetermined time points (0.5, 1, 2, and 4 h), an aliquot of 0.2 ml transport medium (HBSS) was withdrawn from the basolateral chamber and an equal volume of fresh HBSS was replenished. The concentration of Cou-6 in the basolateral chamber was analyzed by a spectral scanning multimode reader. The apparent permeability coefficient (P_app_) was calculated using Eq. (2):

(2) $$P_{\mathrm{app}}=Q/{\mathrm{AC}}_{0}T$$

where Q was the accumulative amount of Cou-6 in the basolateral chamber, A was the membrane growth area, C_0_ was the initial concentration of Cou-6 in the apical chamber and T was the duration time.

After 4 h of incubation, the transport medium from the apical chamber and basolateral chamber were collected and then dialyzed against Pluronic F127 solution (0.1%, *w*/*v*) to remove the salts in the transport medium. After 24 h of dialysis, the samples were then prepared for TEM observation. To validate the integrality of the Caco-2 cell monolayers during the incubation, the TEER values at predetermined time points were measured. The Caco-2 cell monolayers were cut along with the insert membrane from the transwell plate. The tight junctions (TJs) of the Caco-2 cell monolayers were fluorescently stained with Anti-ZO-1/Cy3, and then observed by a confocal laser scanning microscopy (CLSM, FV3000, Olympus, Japan).

To further explore the transport mechanism of SME, Caco-2 cell monolayers were first incubated with C6-SME (100 ng/mL Cou-6) for 2 h. Subsequently, the cell monolayers were washed thrice with fresh HBSS and both the apical chamber and basolateral chamber were added fresh HBSS containing different transport inhibitors, including bafilomycin A1 (150 nM), brefeldin A (25 μg/mL), monensin (32.5 μg/mL) and lomitapide (10 μM). After 4 h of incubation at 37 °C, the amount of C6-SME in the apical chamber and basolateral chamber was measured by a spectral scanning multimode reader (Varioskan Flash, Thermo Scientific, USA). All the experiments were performed in triplicate.

### 2.16 Intracellular colocalization in Caco-2 cells

Caco-2 cells were seeded at a density of 2.0 × 10^5^ cells/well into glass-bottomed 20-mm cell culture dishes. After 36 h of culture, cells were incubated with C6-SME at 37 °C for 4 h. Subsequently, the cells were washed thrice with cold PBS (pH 7.4) and stained with cell trackers (Lyso-Tracker, ER-Tracker, and Golgi-Tracker) according to the manufacturer's protocol. The cells were observed by CLSM.

### 2.17 Cytotoxicity study

Evaluating cytotoxicity is an essential first step to determine the potential antitumor efficacy and safety of a drug candidate or innovative delivery system. The cytotoxicity of drug-loaded SME was evaluated on 4 T1, MCF-7, and HepG2 cells by MTT assay. Briefly, 4 T1, MCF-7 and HepG2 cells were seeded at a density of 7.0 × 10^3^ cells/well into 96-well plates. After 24 h of culture, the culture medium was removed and the cells were washed thrice with PBS (pH 7.4). Subsequently, cells were incubated with DHA-loaded microemulsion, DCC-loaded microemulsion, DSC-loaded microemulsion, or corresponding drug solutions at various concentrations of DHA (0.2, 2, 20, 40, 60, 80, 100, and 200 nmol/mL) at 37 °C for 24 h. Meanwhile, the Caco-2 cell model was employed to evaluate the intestinal safety of blank SME. Caco-2 cells were seeded at a density of 1.0 × 10^4^ cells/well into a 96-well plate. After 36 h of culture, the cells were incubated with blank microemulsion (4.72, 47.22, 472.21, 944.42, 1416.64, 1888.85, 2361.06, and 4722.12 μg/mL) at 37 °C for 24 h. Following incubation of drug-loaded SME and blank SME, each well was incubated with 10 μL MTT solution (5 mg/mL) for 4 h and the medium was removed. For dissolving the formazan crystals, each well was added 150 μL DMSO, and the absorbance values were measured at 490 nm by a spectral scanning multimode reader. All the experiments were performed in quintuplicate. Cell viability was calculated using Eq. (3):

(3) $$\text{Cell viability}\;\left(\%\right)=\left[\left(A_{T}-A_{B}\right)/\left(A_{U}-A_{B}\right)\right]\times 100$$

where A_T_, A_U_, and A_B_ were the absorbance of the incubated groups, the unincubated cells, and the blank medium, respectively. Half maximal inhibitory concentration (IC_50_) was calculated using Graph Pad Prism software.

### 2.18 Cell apoptosis study

To investigate the induction of cellular apoptosis by drug-loaded SME, 4 T1 cells were seeded at a density of 1.0 × 10^5^ cells/well into a 6-well plate. After 24 h of culture, the culture medium was removed and the cells were washed thrice with PBS (pH 7.4). Subsequently, cells were incubated with DHA, DHA-SME, DCC-SME, or DSC-SME (DHA equivalent concentration: 20 nmol/mL) at 37 °C for 24 h. Following this incubation, the cells were washed twice with PBS (pH 7.4), trypsinized, collected. Then, the cells were washed twice with cold PBS (pH 7.4) and resuspended in 100 μL of binding buffer. Finally, the cells were stained with FITC Annexin V and PI according to the manufacturer's protocol, and subsequently subjected to analysis by FCM. All the experiments were performed in triplicate.

### 2.19 Intracellular ROS generation

4 T1 cells were seeded at a density of 1.0 × 10^4^ cells/well into a 24-well plate. After 24 h of culture, the culture medium was removed and the cells were washed thrice with PBS (pH 7.4). Subsequently, cells were incubated with DHA, DHA-SME, DCC-SME, or DSC-SME (DHA equivalent concentration: 20 nmol/mL) at 37 °C for 48 h. Following this incubation, the cells were washed thrice with PBS (pH 7.4) and then incubated with 500 μL of DCFH-DA (10 μM) for 20 min. Finally, the cells were washed thrice with PBS (pH 7.4) and observed by HCS.

### 2.20 Intracellular GSH depletion

4 T1 cells were seeded at a density of 1.0 × 10^5^ cells/well into a 6-well plate. After 24 h of culture, the culture medium was removed and the cells were washed thrice with PBS (pH 7.4). Subsequently, cells were incubated with DHA, DHA-SME, DCC-SME, or DSC-SME (DHA equivalent concentration: 50 nmol/mL) at 37 °C for 24 h. Following this incubation, the cells were harvested to assess intracellular GSH depletion levels using a reduced GSH assay kit according to the manufacturer's protocol, and subsequently subjected to analysis by a spectral scanning multimode reader. All the experiments were performed in triplicate.

### 2.21 Biodistribution in the intestinal tracts and mesenteric lymph nodes

To determine the oral biodistribution of SME in the intestinal tracts and mesenteric lymph nodes (MLNs), a single dose of 2 mg/kg DiR or DiR-SME was orally administered to ICR female mice. At predetermined time points (0.5, 1, 2, and 4 h), the mice were euthanized, and the intestinal tracts and MLNs were collected. Then, fluorescence images of the intestinal tracts and MLNs were taken by an In-Vivo Imaging System (Xtreme, Bruker, Karlsruhe, Germany). All the experiments were performed in triplicate.

To evaluate the uptake of SME in the intestinal tracts and MLNs, Cou-6 or C6-SME were orally administered to ICR female mice at a dose of 4 mg/kg. After 2 h, the mice were euthanized, and intestinal segments (including the duodenum, jejunum, ileum, and colon, approximately 1 cm each) and MLNs were collected. Then, the intestinal segments were fixed with 4% paraformaldehyde, stained with DAPI, and observed by CLSM. As for MLNs, Anti-ZO-1/Cy3 and DAPI were used to stain the intercellular TJs and cell nuclei, respectively.

To further investigate the intestinal lymphatic transport of SME in vivo, mice were intraperitoneally administered 3 mg/kg CHX to block lymphatic transport. One hour post injection, Cou-6 or C6-SME were orally administered to mice at a dose of 4 mg/kg. After 2 h, intestinal segments (including the duodenum, jejunum, ileum, and colon, approximately 1 cm each) and MLNs were collected and examined by CLSM.

### 2.22 Pharmacokinetic study

Twenty male SD rats were distributed randomly into four groups (*n* = 5 per group) using a computer-generated random number sequence. DHA (highly dispersed in 0.5% *w*/*v* carboxymethyl cellulose sodium aqueous solution), DHA-SME, DCC-SME, and DSC-SME (equivalent to 20 mg/kg DHA) were oral administrated. At the fixed time points of 0.167, 0.333, 0.5, 0.75, 1, 2, 4, 6, 8, 12, and 24 h, 0.3 mL of blood samples were collected into 1.5 ml heparinized centrifuge tubes from the fossa orbitalis vein, and were centrifuged at 7,000 rpm for 10 min to obtain the plasma. All plasma samples were stored at −80 °C until HPLC–MS/MS analysis. The HPLC-MS/MS method was described in the “Assay analysis” section. The pharmacokinetic parameters including maximum plasma concentration (C_max_), time of the maximum concentration (T_max_), area under the plasma level-time curve (AUC_0-t_), and mean residence time (MRT_0-t_) were calculated using the Drug and Statistics 2.0 program.

To investigate the intestinal lymphatic transport of SME in vivo, an oral pharmacokinetic study was further performed in the presence of CHX. The SD rats were pretreated with 3.0 mg/kg CHX intraperitoneally.(Ye et al., 2021) After 1 h, DHA and DHA-SME (equivalent to 20 mg/kg DHA) were orally administered. Then, at the fixed time point of 0.167, 0.333, 0.5, 0.75, 1, 2, 4, 6, and 8 h, 0.3 mL of blood samples were collected and processed as described above.

### 2.23 In vivo antitumor efficacy

To evaluate the antitumor efficacy of drug-loaded SME against orthotopic 4 T1 breast tumors, 4 T1 cells (1.0 × 10^6^ cells/mouse) were subcutaneously injected in the mammary fat pad of female BALB/c mice, and the orthotopic 4 T1 tumor models were ready for treatment when the tumor volume reached approximately 100 mm^3^. 4 T1 tumor-bearing mice were randomly assigned to five groups (*n* = 7 per group) using a computer-generated random number sequence and oral administrated with normal saline, DHA, DHA-SME, DCC-SME and DSC-SME (equivalent to 45 mg/kg DHA). All the 4 T1 tumor-bearing mice were oral administered every day for a total of fifteen doses. The tumor volume and the body weight were recorded every 2 days. All tumor measurements and subsequent statistical analyses were performed by investigators blinded to group allocation. Tumor volume was calculated using Eq. (4):

(4) $$\text{Tumor volume}=a\times b^{2}/2$$

where a and b were the length and width of the tumors, respectively.

After the last treatment, all the mice were euthanized and the plasma was collected. The tumors and major organs (heart, liver, spleen, lungs, kidney, stomach, duodenum, and colon) were harvested, cleaned, weighed, photographed, and fixed with 4% paraformaldehyde. Tumor burden was calculated using Eq. (5):

(5) $$\text{Tumor burden}\;\left(\%\right)=W_{t}/W_{m}\times 100$$

where W_t_ and Wm were the weight of excised tumor and tumor-bearing mouse at the endpoint, respectively.

To examine the antitumor efficacy, terminal deoxynucleotidyl transferase dUTP nickend labelling (TUNEL), Ki67, CD31, and hematoxylin & eosin (H&E) staining on the treated tumors were performed and the levels of TNF-α, IL-6, and VEGF in plasma samples were detected with ELISA kits according to the manufacturer's protocol. Part of the harvest tumor tissues were thoroughly homogenized and centrifuged at 3500 rpm for 10 min, and the supernatant was collected for determination of GSH depletion levels consistent with the process for cells. For in vivo safety evaluation, the major organs were stained with H&E and the plasma samples were diluted for analysis of hepatorenal function including serum aspartate aminotransferase (AST), alanine aminotransferase (ALT), alkaline phosphatase (AKP), creatinine (CRE) and blood urea nitrogen (BUN).

Next, to evaluate the antitumor efficacy of drug-loaded SME against tumor metastasis, the orthotopic 4 T1 tumor models with tumor sizes of approximately 100 mm^3^ were established as above and randomly assigned to five groups (*n* = 5 per group) using a computer-generated random number sequence, and then oral administrated with normal saline, DHA, DHA-SME, DCC-SME and DSC-SME (equivalent to 45 mg/kg DHA). All the 4 T1 tumor-bearing mice were oral administered every day for a total of fifteen doses. The tumor volume and the body weight were recorded every 2 days until the 28th day. All tumor measurements and subsequent statistical analyses were performed by investigators blinded to group allocation. Tumor volume and tumor burden were calculated by the above equation. Afterward, all the mice were euthanized and dissected to harvest the tumors and major organs. The tumors, heart, liver, spleen, lungs, and kidney were cleaned, weighed, and photographed. Lungs were divided for separate analyses: one portion was fixed for H&E staining, while another was stained wtih Bouin's dye solution to assess metastasis nodules.

### 2.24 Statistical analysis

All the data are expressed as the mean ± standard deviation (SD). Groups differences were carried out with Student's *t*-test and one-way analysis of variance (ANOVA). *P* < 0.05 indicated statistically significant, and ns represented no significant differences.

## 3 Results and discussion

### 3.1 Synthesis and characterization of DHA prodrugs

As shown in **Fig. S1–S2**, two lipophilic DHA prodrugs (DCC and DSC) were synthesized by conjugating octadecylamine to DHA via an ester bond and a disulfide bond as linkages, respectively. The disulfide bond was chosen as the linkage because of the overexpressed GSH in tumor microenvironment. Meanwhile, the ester bond was selected as a control for the disulfide bond. The structures of DCC and DSC were confirmed by HRMS and ^1^H NMR (**Fig. S3–S4**). The purities of DCC and DSC were evaluated via HPLC. As shown in Fig. **S5–S6**, the chromatograms of DCC and DSC each displayed a single major peak, corresponding to area purities of 95.88% and 95.68%, respectively. These results indicate a level of purity that is well within the acceptable range for subsequent studies.

### 3.2 Prescription study

**Fig. 1:**
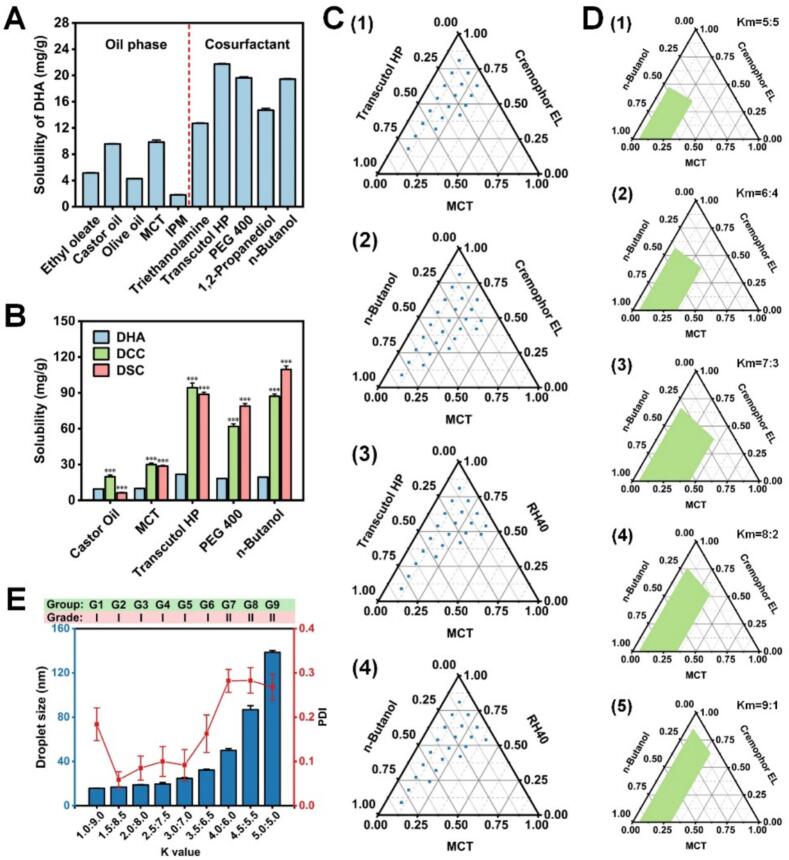
Prescription study and formulation development of SME. Solubilities of (A) DHA and (B) its prodrugs (DCC and DSC) in different oils and cosurfactants. Data are means ± SDs (*n* = 3). ***P < 0.001, compared with DHA. (C) Pseudo-ternary phase diagrams of SME prepared using MCT as the oil, Cremophor EL/RH40 as the surfactant, and transcutol HP/n-butanol as the cosurfactant. (D) Pseudo-ternary phase diagrams of SME prepared using MCT with varying mass ratios of Cremophor EL to n-butanol (Km). (E) The droplet sizes, PDIs, and the grade results of self-microemulsifying ability of formulations with varying mass ratios of surfactant to cosurfactant (K). Data are means ± SDs (*n* = 3).

The oil-water partition coefficient reflects the distribution of drug in the oil and aqueous phases, and is also an important indicator for evaluating the loading efficiency of drug in SME (Cui et al., 2019). The DHA prodrugs (DCC and DSC) are both highly lipophilic compounds with logP values of 12.50 and 12.51 (calculated by ChemBioDraw), repectively. To obtain the optimum drug loading capacity and emulsifying efficiency of SME, solubility study and compatibility study were conducted to identify the appropriate oil, surfactant and cosurfactant. As shown in Fig. 1A, DHA exhibited the highest solubility of 9.56 ± 0.06 mg/g and 9.85 ± 0.30 mg/g in castor oil and MCT among the five oils. Regarding cosurfactants, transcutol HP (21.72 ± 0.11 mg/g) provided the greatest solubilization capability for DHA, followed by PEG400 and n-butanol. Since the selection of oil and cosurfactants should also depend on their drug solubilizing capability for DHA prodrugs, two oils (castor oil and MCT) and three cosurfactants (transcutol HP, PEG400 and n-butanol) were designated for further investigation.

As shown in Fig. 1B, DCC and DSC significantly increased solubility by almost 3-fold compared to DHA in MCT (*p* < 0.001). Therefore, MCT consisting of mono-, di-, and triglycerides was the preferred choice for the oil because of its high affinity for DCC and DSC, easy emulsification properties, and lack of susceptibility to oxidation during long-term storage (Ansari et al., 2023). Moreover, the solubilities of DCC and DSC in transcutol HP, PEG400 and n-butanol were significantly higher than that of DHA (*p* < 0.001). Among them, DCC exhibited the highest solubility of 94.27 ± 3.83 mg/g in transcutol HP, and DSC has the highest solubility of 109.60 ± 2.92 mg/g in n-butanol. Hence, transcutol HP and n-butanol were chosen as the cosurfactants for compatibility study.

The compatibility study was used to further screen surfactants. Six nonionic surfactants were considered as candidates since they were generally safe and acceptable for oral formulation and had been used in several marketed formulations (Rani et al., 2019). The detailed grading results of the state of the microemulsion were shown in **Table S1**. Varying mass ratios of MCT and two nonionic surfactants (cremophor EL and RH40) created a superior microemulsion than other prescription combinations. Meanwhile, the compatibility results of surfactants and cosurfactants were shown in **Table S2**. The compatibility letters of six surfactants and two cosurfactants (transcutol HP and n-butanol) were all “P”, indicating that transcutol HP and n-butanol were all compatible with the selected six nonionic surfactants. In summary, cremophor EL and RH40 were chosen as the surfactants for further study.

### 3.3 Formulation development

The construction of the pseudo-ternary phase diagram is essential for evaluation of the self- microemulsifying ability of SME formulation and establishment of the optimal ratio of the components of the formulations. As shown in Fig. 1C, the combination of MCT, cremophor EL and n-butanol formed the largest self-microemulsification region among all phase digagrams, demonstrating a strong emulsifying ability. Then, five various formulations with surfactant/cosurfactant mass ratios (Km) of 5:5, 6:4, 7:3, 8:2, and 9:1 were tested. When the Km increased from 5:5 to 7.3, the self-microemulsification region was found to be increased and the largest self-microemulsification region was observed with the Km of 7:3 (Fig. 1D). This could be explained that more amount of cremophor EL could effectively reduce the interfacial tension as well as cremophor EL diffusion into the aqueous phase promotes the formation of emulsion droplets (Patel and Sawant, 2019). However, as the Km increased from 7:3 to 9:1, the self-microemulsification region gradually decreased. This could be attributed that more amount of n-butanol could increase the aqueous solubility of MCT and cause the expansion of interfacial film (Patel et al., 2019). Based on the above results, a cremophor EL to n-butanol mass ratio of 7:3 was selected for further optimization and development of SME.

For the selected Km of 7:3, nine various formulations were prepared with varying mass ratios of oil to surfactant/cosurfactant mixtures in the range from 1:9 to 5:5. The state of the microemulsion and the droplet size for nine varying formulations (G1-G9) are shown in Fig. 1E. As the surfactant/cosurfactant concentration decreased, the droplet size and PDI of microemulsion gradually increased, except G1 (K = 1:9). The small droplet size (∼16 nm) with a high PDI value (> 0.150) in G1, which showed an inhomogeneous distribution of droplets. Additionally, the grade results of self-microemulsifying ability of formulations with high surfactant/cosurfactant concentration (G1 to G6) were “I”. The results could be explained that high surfactant/cosurfactant concentration was beneficial for stabilizing and compressing the interfacial film at the oil/water interface, resulting in a decrease in the droplet size. Taken together, G2 was selected as the optimized formulation based on droplet size, PDI, and self-microemulsifying ability. MCT (oil), cremophor EL (surfactant) and n-butanol (cosurfactant) in the proportion of 15:59.5:25.5 (w:w:w) was selected as the optimal prescription of SME for further studies.

### 3.4 Characterization of drug-loaded SME

**Fig. 2:**
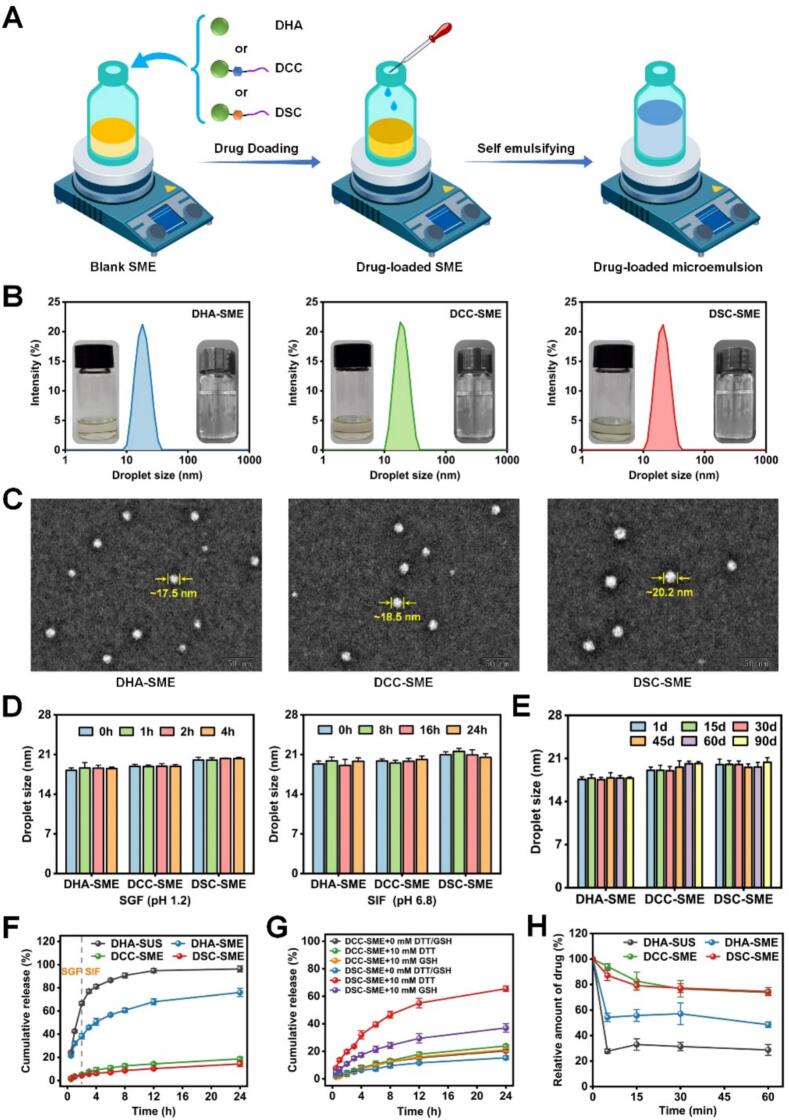
Characterization, release profiles, and stability of drug-loaded SME. (A) The preparation schematic illustration of drug-loaded SMEs and their microemulsion. (B) Droplet size distribution, unemulsified (left) and microemulsion (right) appearance of DHA-SME, DCC-SME, and DSC-SME. (C) Transmission electron microscopy (TEM) images of DHA-SME, DCC-SME, and DSC-SME. (D) Medium stability of DHA-SME, DCC-SME, and DSC-SME after incubation in simulated gastric fluid (SGF, pH 1.2) and simulated intestinal fluid (SIF, pH 6.8). Data are means ± SDs (*n* = 3). (E) Storage stability of DHA-SME, DCC-SME, and DSC-SME after 90 days of storage at room temperature 4 °C. Data are means ± SDs (n = 3). (F) In vitro release profiles of DHA-SUS, DHA-SME, DCC-SME and DSC-SME in SGF (pH 1.2) for 2 h and SIF (pH 6.8) for 22 h. Data are means ± SDs (n = 3). (G) The DHA release profiles of DHA-SUS, DHA-SME, DCC-SME and DSC-SME in PBS with 10 mM DTT or GSH for 24 h. Data are means ± SDs (n = 3). (H) Relative amount of drug in DHA-SUS, DHA-SME, DCC-SME and DSC-SME solubilized in aqueous phase during the lipolysis of 60 min (n = 3).

As shown in Fig. 2A-B, DHA-SME, DCC-SME, and DSC-SME were efficiently prepared by simply mixing the optimized quantity of each drug (DHA, DCC, or DSC) with the blank SME. At room temperature, DHA-SME, DCC-SME, and DSC-SME all appeared as clear, light-yellow, and viscous liquids. Furthermore, the three drug-loaded SMEs exhibits rapid self-emulsification, forming colorless, transparent microemulsions spontaneously within one minute upon aqueous dilution. The droplet size, PDI, zeta potential, equilibrium solubility, drug loading capacity, and EE of the three drug-loaded SMEs and their corresponding microemulsions are shown in **Table S3**. The droplet size of all three microemulsions was ∼20 nm with a PDI of <0.1, and a zeta potential of around −2 mV. As shown in Fig. 2C, the TEM images showed that three microemulsions presented spherical shapes with uniform droplet size distribution (∼ 20 nm), well consistent with the droplet size results. The molar drug loading capacity of DCC and DSC were 1.17- and 1.83-fold higher than that of DHA in the same composition as that of SME, which confirmed that lipophilic prodrugs of DHA could remarkably improve its affinity and compatibility with the oil phase of SME. Moreover, the optimal SME loaded with DHA or prodrugs had a similar EE (> 99%). Considering the initial contact with gastric acid post-administration, the stability of drug-loaded SMEs upon dilution in diluted hydrochloric acid (pH 1.2) was assessed (Sharma et al., 2023). As shown in **Fig. S7**, no significant changes (*P* > 0.05) in droplet size were observed for the three drug loaded SMEs after dilution at a volume ratio of 1:5 to 1:500, indicating that the prepared DHA-SME, DCC-SME, and DSC-SME had good dilution stability.

### 3.5 Medium stability and storage of DSC-SME

Given the challenging gastrointestinal milieu including low pH and enzymatic activity that orally delivered SME encounter, the gastrointestinal medium stability is imperative for ensuring sufficient absorption and maintaining intended efficacy. The stability of DHA-SME, DCC-SME, and DSC-SME was first assessed in simulated gastrointestinal fluids. As shown in Fig. 2D and S8, no significant changes in droplet size were observed for three formulations after 4 h in SGF (pH 1.2) or 24 h in SIF (pH 6.8) at 37 °C (*P* > 0.05). Moreover, stability was also evaluated in PBS (pH 7.4) to confirm compatibility with the aqueous environment used in subsequent cell-based assays. Following 24 h of incubation, the droplet sizes of three formulations remained unchanged from the initial values. It can be concluded that DHA-SME, DCC-SME and DSC-SME are sufficiently robust to maintain their nanostructure during gastrointestinal transit, a key prerequisite for ensuring their subsequent uptake in an intact form.

To evaluate storage stability, the appearance of DHA-SME, DCC-SME and DSC-SME was observed and the droplet size of their corresponding microemulsions was measured over a 90 days period at room temperature and 4 °C. All formulations exhibited no visible changes, such as phase separation or precipitation, and maintained their clarity. Furthermore, droplet size analysis (Fig. 2E **and S9**) revealed no significant changes (P > 0.05). These results collectively indicated that DHA-SME, DCC-SME and DSC-SME were stable under the tested conditions for at least 90 days.

### 3.6 In vitro release assay

The in vitro release profiles of DHA-SME, DCC-SME, and DSC-SME in a continuous alternative release medium (from SGF to SIF) are shown in Fig. 2F. The release of DHA was slower from DHA-SME than from DHA-SUS upon sequential incubation in SGF (2 h) and SIF (22*h*) at 37 °C. More than 90% of DHA was released from DHA-SUS after 8 h, whereas only about 60% of DHA was released from DHA-SME, suggesting that the SME formulation effectively protected DHA from premature release in the gastrointestinal tract. Compared with DHA-SME, both DCC-SME and DSC-SME showed minimal drug release (< 20% cumulative release at 24 h). This can be attributed to the high lipophilicity of the DHA prodrugs, which enhances their compatibility with and encapsulation into the oil phase of SME. Additionally, a burst release of DHA from DHA-SME was observed in SGF at 0.5 h, with over 20% of the drug release cumulatively. This likely results from two factors. The first is the poor affinity between DHA and the SME formulation, which may lead to a portion of DHA being physically adsorbed or only loosely associated with the nanocarrier surface. The second is the relatively low molecular weight of DHA, which, compared to larger prodrug molecules, may allow it to diffuse rapidly out of the nanocarrier matrix. Consequently, the sustained drug release of the prodrug-loaded SME ensured that drug was absorbed orally primarily in the form of a microemulsion rather than as the free drug form.

Given that drug release from the prodrug is crucial for its antitumor efficacy, we further investigated the reduction-responsive release behavior of DSC-SME. To simulate the reductive tumor microenvironment, PBS containing 10 mM of a reducing agent (DTT or GSH) was employed as the release medium. As shown in Fig. 2G, DHA release from DCC-SME was comparable under all tested conditions (23.9% with DTT, 21.1% with GSH, and 20.4% without reducing agent), confirming that the CC bond is insensitive to reductive environments. In contrast, DSC-SME exhibited substantial reduction-triggered DHA release, with cumulative rates of 65.5% and 37.0% at 24 h in the presence of DTT and GSH, respectively, compared to only 15% in the absence of a reducing agent. The differential release kinetics between DTT and GSH can be attributed to the fact that DTT contains two sulfhydryl groups, providing a stronger reducing capacity for disulfide bond cleavage, whereas GSH possesses only a single sulfhydryl group, leading to slower cleavage (Liu et al., 2025). Collectively, these results demonstrate that the disulfide bond in DSC enables its conversion to active DHA under reductive conditions, representing a critical validation step for the prodrug design.

### 3.7 In vitro lipolysis study

Upon intestinal transport, the lipid constituents of SMEs undergo lipolysis. During this digestive process, the drugs encapsulated within SMEs may either become solubilized in the newly formed colloidal aqueous phase or precipitate within the gastrointestinal tract, thereby affecting drug absorption (Zhang et al., 2023). The aqueous phase partitioning profiles of DHA-SUS, DHA-SME, DCC-SME, and DSC-SME during the 60 min lipolysis process are presented in Fig. 2H. Owing to the low water solubility of DHA, the DHA amount in the aqueous phase of DHA-SUS declined to 27.8% after 5 min lipolysis, suggesting rapid precipitation of DHA following intestinal digestion. In the case of DHA-SME, the aqueous DHA fraction decreased to 54.3% at 5 min, which is attributable to the compatibilization effect conferred by the SME formulation. By contrast, DCC-SME and DSC-SME substantially enhanced drug solubilization, maintaining >70% of the drug in the aqueous phase throughout the lipolysis process. Moreover, the percentage of the solubilized DCC and DSC were both approximatedly 1.5-fold higher than that of DHA-SME after 60 min of digestion. These results demonstrated that the lipophilic prodrug strategy promoted the solubilization of DHA within colloidal structures formed during lipid digestion, such as mixed micelles derived from lipolytic products.

### 3.8 Cellular uptake and endocytosis pathways study

**Fig. 3:**
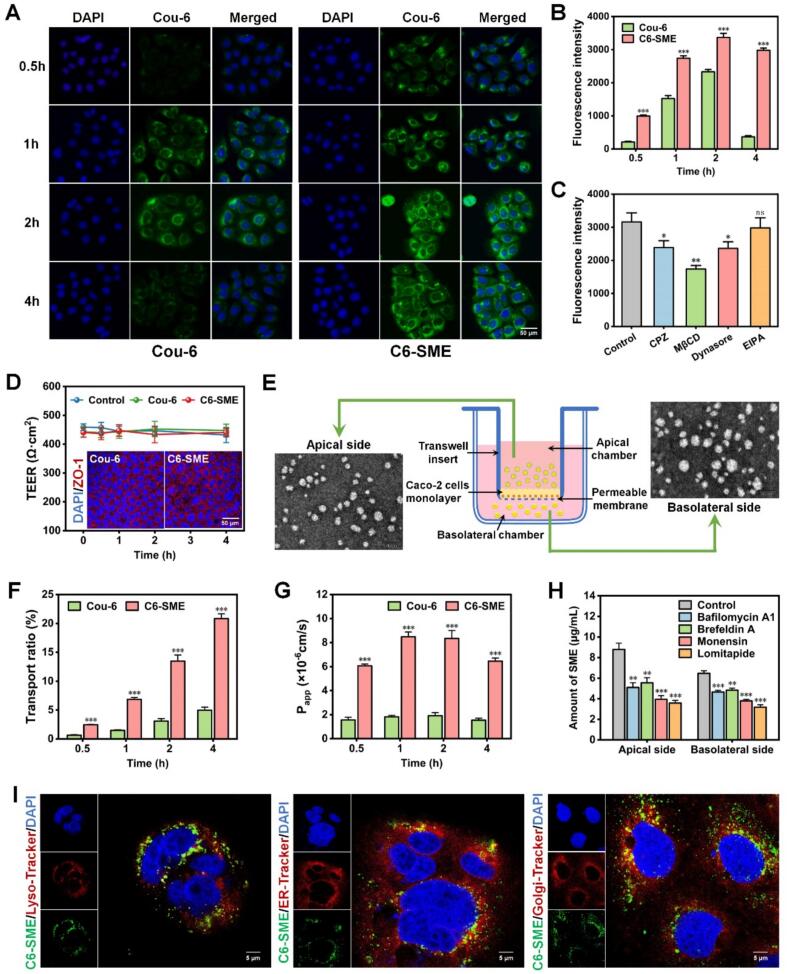
Cellular uptake profile, endocytosis pathway and intracellular transport pathway of SME. (A) HCS images of the cellular uptake of Cou-6 and C6-SME (green signal) in Caco-2 cells after incubation for various time intervals at 37 °C. Cell nuclei were stained with DAPI (blue signal). (B) Flow cytometric detection of fluorescence intensity of Cou-6 and C6-SME in Caco-2 cells after incubation for various time intervals at 37 °C. Data are means ± SDs (*n* = 3). ***P < 0.001, compared with Cou-6. (C) Impacts of various endocytosis inhibitors on the internalization of C6-SME in Caco-2 cells. Data are means ± SDs (n = 3). *P < 0.05, **P < 0.01, ns, no significant difference, compared with control. (D) TEER changes and ZO-1 immunofluorescence imaging of Caco-2 cell monolayers after the transportation of Cou-6 or C6-SME. (E) TEM images of C6-SME in the apical or basolateral chamber after the incubation for 4 h. (F) Transport ratios of Cou-6 and C6-SME at predetermined time points in Caco-2 cell monolayers. Data are means ± SDs (n = 3). ***P < 0.001, compared with Cou-6. (G) P_app_ values of Cou-6 and C6-SME at predetermined time points in Caco-2 cell monolayers. Data are means ± SDs (n = 3). ***P < 0.001, compared with Cou-6. (H) Impacts of various transport inhibitors on the transportation of C6-SME across the Caco-2 cell monolayers via the apical or the basolateral membrane. Data are means ± SDs (n = 3). **P < 0.01, ***P < 0.001, compared with control. (I) CLSM images of the colocalization of C6-SME (green signal) with different organelles (red signal) in Caco-2 cells. Cell nuclei were stained with DAPI (blue signal). The yellow dots in the merged images indicate the colocalization of C6-SME with the different organelles.

The cellular uptake efficiency of SME in Caco-2 cells was evaluated by detecting the intracellular fluorescence signals of Cou-6 employing HCS and FCM, respectively. As shown in Fig. 3A-3B **and S10**, as incubation time was prolonged from 0.5 h to 2 h, the cellular uptake of both Cou-6 and C6-SME in Caco-2 cells gradually increased, demonstrating that their internalization is time-dependent. Compared to the 2-h time point, the cellular fluorescence intensity of the C6-SME group was slightly weaker at 4 h, suggesting that its cellular uptake reached saturation by 2 h. In contrast, cellular fluorescence signal from the Cou-6 group was nearly undetected at the 4-h mark. Notably, the cellular fluorescence intensity of C6-SME was markedly higher than that of Cou-6 (*P* < 0.001), indicating that SME could dramatically elevate the cellular uptake efficacy of encapsulated drugs and protect drugs from degradation.

To gain insight into the cellular internalization mechanism of SME, a panel of endocytosis inhibitors was used: CPZ (clathrin-mediated), MβCD (caveolae/lipid raft-mediated), dynasore (dynamin-dependent), and EIPA (macropinocytosis). As shown in Fig. 3C, the cellular uptake of SME was significantly inhibited by CPZ (to 75.41%) and MβCD (to 55.00%), suggesting the involvement of both clathrin- and caveolae/lipid raft-mediated endocytosis pathways in Caco-2 cells. Dynasore pretreatment also significantly inhibited SME uptake (*P* < 0.05), which is consistent with its role as an inhibitor of dynamin—a GTPase required for both clathrin- and caveolae/lipid raft-mediated endocytosis (Ahmed et al., 2023). In contrast, EIPA did not significantly inhibit SME uptake, ruling out macropinocytosis as a major pathway. While these pharmacological inhibitor data point to the involvement of clathrin- and caveolae/lipid raft-mediated endocytosis, it should be noted that inhibitors are not entirely specific for a single endocytic pathway. Moreover, endocytic pathways are not mutually exclusive; multiple pathways can operate simultaneously or compensate for one another when a single pathway is blocked. Collectively, these results suggest that caveolae/lipid raft- and clathrin-mediated endocytosis, rather than macropinocytosis, are the primary pathways involved in SME internalization in Caco-2 cells. In addition, particle size is the dominant factor governing the cellular internalization of nanocarriers. In general, nanoparticles smaller than 50 nm tend to enter cells via caveolae/lipid raft-mediated endocytosis, while those in the range of 50–200 nm are preferentially internalized through clathrin-mediated endocytosis. Larger particles (> 200 nm) may rely on macropinocytosis or phagocytosis. Besides, surface charge of nanocarriers also influences the uptake pathway, being negatively charged nanocarriers more easily taken up into cells by caveolae/lipid raft-mediated endocytosis while positively charged nanocarriers seem to prefer clathrin-mediated endocytosis (Manzanares and Ceña, 2020). In the present study, the smaller particle size (< 50 nm) and slightly negative surface charge (−2 mV) of SME fall within the favorable ranges for cellular uptake via caveolae/lipid raft-dependent, further supporting the results of endocytosis pathways.

### 3.9 Transepithelial transport study using the Caco-2 cell monolayers

The TEER value and AKP activity of the intestinal epithelial cell model help reflect the tightness and barrier integrity of the Caco-2 cell monolayers. As shown in Fig. 3D, the TEER value of the Caco-2 cell monolayers was approximately 100 Ω·cm^2^ on day 5 post-inoculation. Subsequently, the TEER value exhibited a continuous increase, eventually stabilizing at around 500 Ω·cm^2^ by day 21. Concurrently, the ratio of AKP activity between the apical and basolateral chamber (AKP_AP_/AKP_BL_) began to rise from day 5 onward and reached a plateau after day 17. This increase in AKP_AP_/AKP_BL_ indicated the successful differentiation of Caco-2 cells into a specialized intestinal epithelial structure (Hu et al., 2021). Taken together, these results demonstrate the successful establishment of a mature Caco-2 cell monolayer, which is suitable for subsequent transepithelial transport studies.

To assess whether SME could enhance transepithelial transport, the delivery of Cou-6 in its free form versus encapsulated within SME (C6-SME) was compared. As showed in Fig. 3F, both free Cou-6 and C6-SME exhibited time-dependent transport across the Caco-2 cell monolayers. After 4 h of incubation, the transport efficiency of C6-SME reached approximately 20.83%, which was 4.21-fold higher than that of free Cou-6 (approximately 4.95%). Moreover, the apparent permeability coefficient (P_app_) of free Cou-6 remained consistently low and was significantly lower than that of C6-SME (*P* < 0.001) at all time points (Fig. 3G). The P_app_ value of C6-SME initially increased during the first hour (0.5–1 h), plateaued at 2 h, and subsequently declined from 2 to 4 h. At 2 h, the P_app_ value of C6-SME was 4.37-fold as compared with Cou-6. The transport ratio and P_app_ results of SME were consistent with the cellular uptake efficiency results presented in Fig. 3B, where SME could remark enhance the transepithelial efficiency of encapsulated drugs.

During the entire process of transport experiment, the TEER value of both free Cou-6 and C6-SME were measured and exhibited unchanged compared to PBS (control). Following the transport experiment, the integrity of the TJs in the Caco-2 monolayers was assessed by CLSM. As shown in Fig. 3D, a key TJs protein, the ZO-1 immunofluorescence staining revealed a continuous distribution at cell borders after incubation with either free Cou-6 or C6-SME. The unchanged TEER values and intact ZO-1 staining collectively indicated that the integrity of TJs was not impaired (Bao et al., 2023). Therefore, neither Cou-6 nor C6-SME adversely affected the Caco-2 cell monolayers. This finding suggests that SME was transported primarily via the transcellular pathway rather than the paracellular route. Assessing how transport across intestinal epithelial cell monolayers affects nanoparticles is critical to evaluating their potential for drug protection, controlled release, and targeted delivery. To this end, TEM was employed to determine whether SME remained intact following the transepithelial transport. Fig. 3E shows the morphology of C6-SME in the in the apical and basolateral chamber after 4 h of transportation across the Caco-2 cell monolayers. Microemulsion droplets were observed to have crossed into the basolateral chamber, retaining their spherical structure similar to that in the apical chamber, which proved that SME transported crossing the Caco-2 cell monolayers remained intact. More importantly, following epithelial transport, the droplet size increased from an initial 20 nm to approximately 30 nm. This increase may be related to the chylomicron pathway and possible apolipoprotein assembly during intracellular transport, though this association remains speculative and requires further experimental validation in subsequent research (Mao et al., 2019).

To further elucidate the intracellular transport pathways of SME, the impacts of various intracellular transport inhibitors on the discharge of SME via the apical or basolateral membrane of the Caco-2 cell monolayers were investigated (Fig. 3H). Four inhibitors targeting distinct transport steps were used: Bafilomycin A1, which functions as a lysosome acidification inhibitor to hinder endosome maturation; Brefeldin A, which disrupts endoplasmic reticulum (ER)-to-Golgi transport; Monensin, which inhibits Golgi-to-plasma membrane trafficking; and Lomitapide, which acts as an MTTP inhibitor to block chylomicron assembly. Relative to the control, bafilomycin A1, brefeldin A, and monensin each significantly increased the intracellular accumulation of SME within the Caco-2 cell monolayers (*P* < 0.01). These results suggested that SME exocytosis was a complex process involving an intricate interplay of lysosomal, ER, and Golgi apparatus functions. Meanwhile, following the suppression of chylomicron assembly by lomitapide, SME levels in both chambers decreased significantly (*P* < 0.001), indicating a potential role of this pathway in SME exocytosis from the Caco-2 cell monolayers. Given that the ER/Golgi apparatus route was involved in chylomicron assembly and secretion, the present findings raise the possibility that SME transport may share a similar trafficking pathway. Nevertheless, further studies are needed to confirm the precise mechanism and to rule out alternative explanations.

To validate the proposed intracellular transport pathway of SME, the intracellular co-localization analysis was performed using CLSM. Caco-2 cells were stained for markers of lysosomes, ER and Golgi apparatus to assess their association with internalized SME. Fig. 3I shows the subcellular distribution in Caco-2 cells visualized by CLSM. Cell nuclei are stained blue, specific organelles are labeled red, and C6-SME appears green. In the merged image, yellow puncta indicate co-localization between the organelle and C6-SME. As shown in Fig. 3I, C6-SME fluorescence clearly co-localizes with trackers for lysosomes, ER, and Golgi apparatus, providing visual evidence to support its transport via these intracellular pathways. Via the ER/Golgi pathway, SME are incorporated as lipid components during chylomicron assembly and are subsequently secreted from intestinal epithelial cells into the intestinal lymphatic system (Deng et al., 2022).

### 3.10 Cytotoxicity and cell apoptosis study

**Fig. 4:**
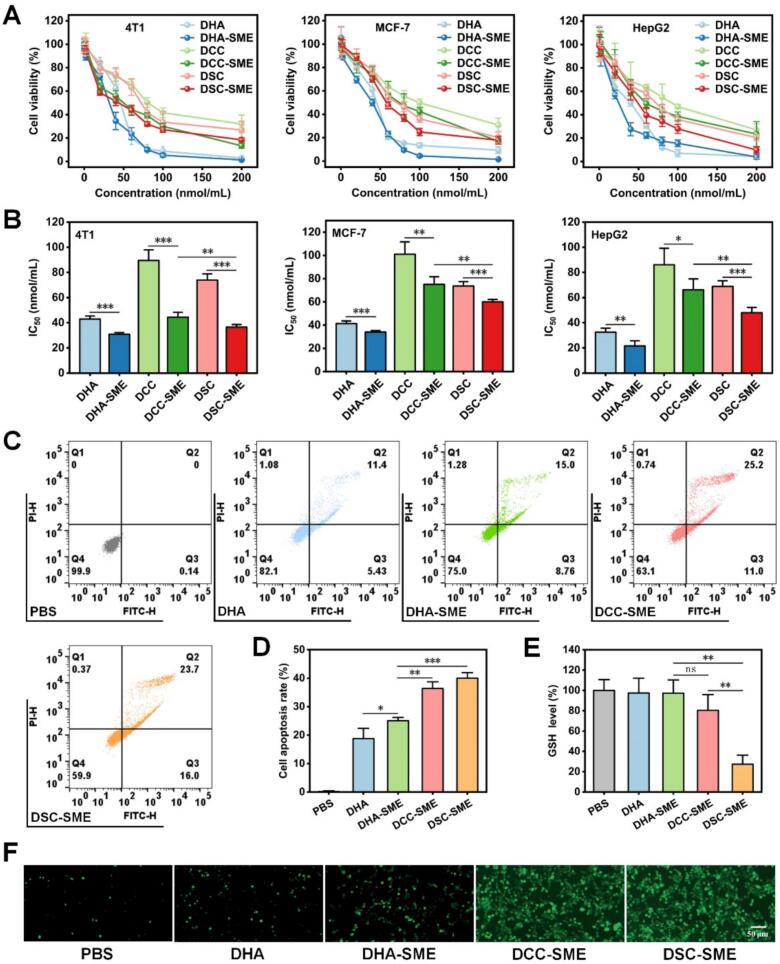
In vitro antitumor efficacy of DSC-SME. (A) Cell viability of 4 T1, MCF-7, and HepG2 cells after treatment with various concentration of DHA, DHA-SME, DCC, DCC-SME, DSC, and DSC-SME for 24 h. Data are means ± SDs (*n* = 5). (B) Cytotoxicity of DHA, DHA-SME, DCC, DCC-SME, DSC, and DSC-SME on 4 T1, MCF-7, and HepG2 cells. Data are means ± SDs (n = 5). (C) Flow cytometric analysis, (D) quantitative analysis of cell apoptosis in 4 T1 cells after treatment with DHA, DHA-SME, DCC-SME, and DSC-SME for 24 h. Data are means ± SDs (*n* = 3). *P < 0.05, **P < 0.01, ***P < 0.001. (E) GSH levels in 4 T1 cells after treatment with DHA, DHA-SME, DCC-SME, and DSC-SME for 24 h. Data are means ± SDs (*n* = 3). **P < 0.01, ns, no significant difference. (F) HCS images of ROS level in 4 T1 cells after treatment with DHA, DHA-SME, DCC-SME, and DSC-SME for 48 h.

The MTT method was applied to determine the cytotoxicity of prodrug-load SME and corresponding drug solutions on two kinds of breast cancer cells (4 T1 cells and MCF-7 cells) and HepG2 human hepatoma cells. As shown in Fig. 4A-4B and **Table S4**, DHA, DHA-SME, DCC, DCC-SME, DSC, and DSC-SME showed potent cytotoxicity against multiple cancer cell lines, with effects depending on DHA concentration (0.2–200 nmol/mL).

The IC_50_ values of drug-loaded SME were significantly reduced compared to corresponding drug solution in three tumor cells (P < 0.05). This stronger cytotoxicity of drug-loaded SME was due to the enhanced cellular internalization of SME. Besides, the DHA prodrugs (DCC and DSC) exhibited higher IC_50_ values than DHA in both the drug solution group and SME group, possibly due to incomplete conversion to the active ingredient, which would limit their efficacy. The active drug DHA exerts potent cytotoxic effects on three kinds cancer cells through multiple interconnected molecular mechanisms, which are closely associated with its unique chemical structure (containing an endoperoxide bridge) and its ability to regulate key cellular signaling pathways. The core cytotoxic mechanisms of DHA mainly include ferroptosis, apoptosis, and autophagy. For prodrug-loaded SME, DSC-SME exhibited stronger cytotoxicity than DCC-SME. It could be due to the rapid release of DHA in the high GSH microenvironment of tumor cells, which is triggered by the breaking of disulfide bonds (Guo et al., 2026). More important, a toxicity study on intestinal epithelial cells is necessary for this novel oral SME formulation. As shown in **Fig. S11**, the cell viability in Caco-2 cells after 24 h of incubation with blank microemulsion (4.72–4722.12 μg/mL) corresponding to DHA concentrations of 0.2–200 nmol/mL was near 100%. The result demonstrated that blank SME did not significantly affect the biological activity of Caco-2 cells at the tested concentrations and time points, confirming its safety for oral administration.

To further explore the antitumor efficacy of the prodrug-loaded SME, apoptosis in 4 T1 cells treated with DHA, DHA-SME, DCC-SME, and DSC-SME (20 nmol/mL) was detected by flow cytometry using an FITC Annexin V and PI dual staining kits. As shown in Fig. 4C-D, the apoptosis rate was 0.15% in the control group. For the treatment groups, the primary mechanism of action was the induction of both early and late apoptosis in 4 T1 cells. The apoptosis rates increased in the order of DHA (18.77%) < DHA-SME (25.03%) < DCC-SME (36.37%) < DSC-SME (40.00%). Among them, the difference between DCC-SME and DSC-SME was not significant, which is consistent with the cytotoxicity results in 4 T1 cells.

### 3.11 Intracellular ROS generation and GSH depletion

Compared to normal tissues, various tumor cells exhibit significantly higher levels of GSH. While GSH concentrations in normal tissues and plasma range from 0.5 to 10 mmol/L, they are over 4-fold higher in tumor cells. This elevated GSH environment is detrimental to antitumor therapy. Conversely, depleting GSH through mechanisms such as disulfide bond cleavage can enhance tumor inhibition. To evaluate the GSH sensitivity of DSC-SME in 4 T1 cells, the intracellular reduced GSH content was measured using a reduced GSH assay kit. As shown in Fig. 4E, there was no significant difference in reduced GSH levels among the DHA, DHA-SME, and DCC-SME groups compared with the control group. The GSH level in the DSC-SME group was 27.25%, which was distinctly lower than those in the DHA-SME and DCC-SME groups. This suggested that the disulfide bonds in DSC-SME were sensitive to GSH and could deplete intracellular GSH, thereby inducing DHA release from the prodrug at the tumor site to exert more potent anti-tumor effects.

DHA possess an intramolecular endoperoxide bridge and the endoperoxide bond can be activated by heme or ferrous iron to produce the cytotoxic reactive oxygen species (ROS) (Li et al., 2026). ROS will exert a toxic effect on cell and affect the growth of cells. Hence, the potent cytotoxicity of DHA could be closed related to its cellular ROS generation. The ROS levels in 4 T1 cells treated with DHA, DHA-SME, DCC-SME, and DSC-SME were detected by HCS using a DCFH-DA ROS assay kit. As shown in Fig. 4F, compared to the PBS group, the green fluorescence signal in 4 T1 cells treated with DHA was stronger, yet it remained weaker than that in the DHA-SME group, which should be attributed to the DHA-induced ROS generation and the superior cellular uptake enabled by the SME formulation. Moreover, both DCC-SME and DSC-SME markedly increased intracellular ROS levels, indicating their enhanced cytotoxicity against tumor cells. This effect was owing to the strong compatibility between the highly lipophilic prodrugs and the lipid cores of SME, which effectively prevented the premature leakage of DHA prior to cellular uptake (Gao et al., 2021).

### 3.12 Biodistribution of SME in the intestinal tract and MLNs

**Fig. 5:**
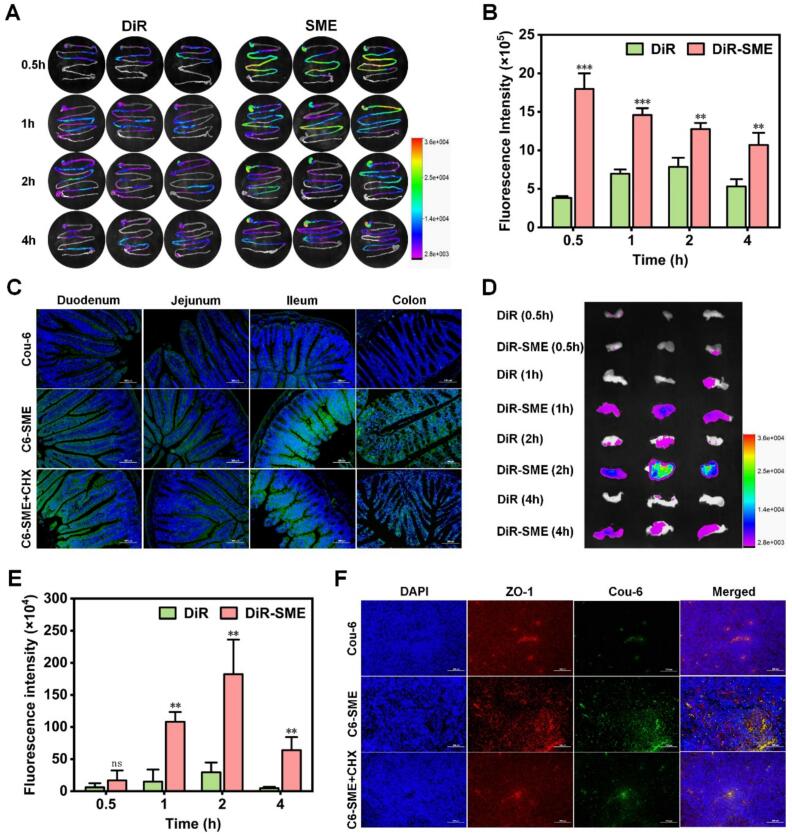
Biodistribution of DiR-SME in the intestinal tract and MLNs. (A) Ex vivo fluorescence images of the distribution of DiR and DiR-SME in the intestinal tract after oral administration at predetermined time points. (B) The quality of DiR and DiR-SME remained in the intestinal tract. Data are means ± SDs (*n* = 3). **P < 0.01, ***P < 0.001, compared with DiR. (C) CLSM images of Cou-6 and C6-SME (green signal) in duodenum, jejunum, ileum, and colon sections after oral administration for 2 h. To block intestinal lymphatic transport, mice were intraperitoneally pretreated with a dose of 3.0 mg/kg CHX 1 h prior to drug administration. Cell nuclei were stained with DAPI (blue signal). (D) Ex vivo fluorescence images of the distribution of DiR and DiR-SME in MLNs after oral administration at predetermined time points. (E) The quality of DiR and DiR-SME remained in MLNs. Data are means ± SDs (n = 3). **P < 0.01, ns, no significant difference, compared with DiR. (F) CLSM images of Cou-6 and C6-SME (green signal) in MLNs after oral administration for 2 h. To block intestinal lymphatic transport, mice were intraperitoneally pretreated with a dose of 3.0 mg/kg CHX 1 h prior to drug administration. Cell nuclei and intercellular tight junction were stained with DAPI (blue signal) and Anti-ZO-1/Cy3 (red signal).

To assess the in vivo biodistribution within the intestinal tract and MLNs, DiR solution or DiR-SME was administered orally. At predetermined time points (0.5, 1, 2, and 4 h), the entire intestinal tract and MLNs were harvested and subjected to ex vivo fluorescence imaging using an In-Vivo Imaging System. As shown in Fig. 5A, fluorescence signals of free DiR and DiR-SME were detectable throughout all segments of the intestinal tract at the 2-h time point post-administration. By the 4-h time point, DiR-SME remained widely distributed throughout the gastrointestinal tract, whereas free DiR exhibited a marked reduction in fluorescence, particularly in the anterior intestinal segments. Quantitative analysis of the intestinal fluorescence signals further showed that the fluorescence intensity peaked at 1 h for DiR-SME and subsequently declined over time (Fig. 5B). Moreover, the fluorescence intensity of the DiR-SME group was consistently higher than that of the free DiR group at all time point examined (0.5, 1, 2, and 4 h). The above data confirmed that SME enhanced mucoadhesion and prolonged intestinal residence time, thereby potentially improving bioavailability. Furthermore, detection of the formulation in the MLNs could further validated lymphatic transport. Fig. 5D shows pronounced accumulation of DiR-SME in the MLNs, while only a minimal signal was evident in the free DiR group, indicating limited lymphatic transport. As shown in Fig. 5E, the fluorescence intensity in the MLNs for the SME group rose to a maximum at 2 h (approximately 6-fold greater than the free DiR group) and then gradually decreased. These results confirmed the lymphatic targeting capability of the prepared SME, which was capable of significantly augmenting the distribution of encapsulated drugs to MLNs.

To further verify the biodistribution of SME, sections of intestinal segments (duodenum, jejunum, ileum, and colon) and of the MLNs were prepared and examined by CLSM. Meanwhile, this was complemented by a mechanistic experiment in which mice were pretreated with cycloheximide (i.p.) to block chylomicron flow prior to dosing, testing the hypothesis that this pathway mediates the increased MLN accumulation of SME. As shown in Fig. 5C and F, cell nuclei were stained with DAPI (blue), and the green fluorescence corresponded to free Cou-6 or C6-SME, respectively. Consistent with the ex vivo fluorescence images, the C6-SME group exhibited a markedly higher fluorescence signal than the free Cou-6 group in all intestinal segments and the MLNs. Furthermore, pretreatment with CHX (C6-SME + CHX) increased fluorescence on the surface of duodenal, jejunal, and ileal villi while decreasing it in lacteals and MLNs versus C6-SME alone. This suggested that by inhibiting chylomicron flow, CHX impeded the entry of C6-SME into the lymphatic system (lacteals), thereby causing its accumulation at the intestinal surface and reducing its subsequent trafficking to the MLNs (Al Nebaihi et al., 2023). Taken together, these results suggested that SME could enhance drug permeation through the intestinal epithelial cells and promote drug accumulation within the lymphatic system via the chylomicrons pathway.

### 3.13 Pharmacokinetic study and intestinal lymphatic transport inhibition study of DSC-SME

**Fig. 6:**
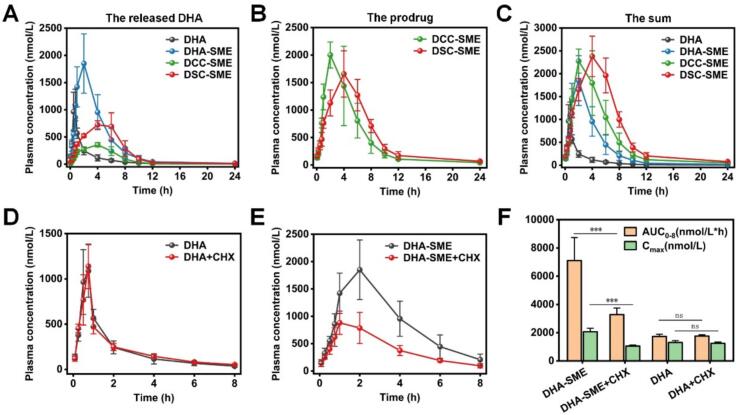
Pharmacokinetic profiles and intestinal lymphatic transport inhibition result of DHA suspension, DHA-SME, DCC-SME, and DSC-SME. Plasma concentrations-time curves of (A) the released DHA, (B) the prodrug, and (C) the sum of the released DHA and prodrug after a single oral administration in rats. Data are means ± SDs (*n* = 5). Plasma concentrations-time curves of DHA after a single oral administration of (D) DHA suspension and (E) DHA-SME in rats pretreated with or without cycloheximide. Data are means ± SDs (n = 5). (F) Some pharmacokinetic parameters of DHA in the intestinal lymphatic transport inhibition study. Data are means ± SDs (n = 5). ^⁎⁎⁎^*P* < 0.001, ns, no significant difference.

The pharmacokinetic behaviors of DHA suspension, DHA-SME, DCC-SME, and DSC-SME in SD rats were detected by HPLC-MS/MS after a single oral administration. The main pharmacokinetic parameters of the four administration groups are presented in **Table S5**, and the molar concentration-time curves of the released DHA, the prodrugs, and the sum are shown in Fig. 6A-C. The C_max_ (2066.26 ± 244.61 nmol/L) and AUC_0∼24_ (7909.65 ± 1793.26 nmol/L*h) of DHA in the DHA-SME group were 1.58- and 4.17-fold greater than those in the DHA suspension group (free DHA), respectively. Orally administered DHA suspension was rapidly absorbed (T_max_ = 0.65 h). In contrast, DHA-SME showed a delayed T_max_ of 1.60 h and a prolonged mean residence time (MRT_0__–__24_ = 3.85 h vs. 2.92 h for the DHA suspension). This sustained release profile is consistent with the in vitro release result (Fig. 2F), suggesting that the SME formulation showed a slow rate of absorption and reduced the elimination rate of DHA, thereby extending its systemic exposure. These results indicated that the SME formulation could markedly enhance the oral absorption of DHA. The superior pharmacokinetic profile of DHA-SME is owing to a combination of mechanisms: (i) enhanced GI solubility of DHA; (ii) improved stability through protection from GI degradation; (iii) facilitated cellular uptake due to good biocompatibility; and (iv) lymphatic targeting via the chylomicron pathway, which avoids first-pass hepatic metabolism (Li et al., 2025). In addition, prodrug-loaded SMEs (DCC-SME and DSC-SME) exhibited superior pharmacokinetics compared to DHA-SME. Their AUC_0__–__24_ values (the sum) were 1.64- and 2.14-fold higher, respectively, accompanied by further prolongations in T_max_ and MRT_0__–__24_, which indicated that the lipophilic prodrug combined with SME could further improve the oral bioavailability compared to DHA-SME. The improvement can be attributed to two key factors. Firstly, the good compatibility between lipophilic prodrug and the SME formulation ensures stable encapsulation and minimizes drug leakage. Secondly, the lipid-based formulation is particularly effective in shuttling lipophilic drugs (logP >5) into the lymphatic system, a route that bypasses first-pass hepatic metabolism and significantly enhances systemic drug exposure (Zhang et al., 2023). As shown in **Table S5** and Fig. 6A-B, following the C_max_ of the prodrugs in the prodrug-loaded SMEs group, the concentration of released DHA reached its own C_max_ within 2 h. This temporal profile suggested that a portion of both DCC and DSC prodrugs undergo hydrolysis to the active parent drug during systemic circulation. Furthermore, the AUC_0__–__24_ of DCC and DSC in their respective SME groups was higher than that of DHA suspension, suggesting that a substantial fraction of these prodrugs was not released from the SME and remained intact in the systemic circulation without rapid hydrolysis, which would be beneficial to the tumor accumulation and therapeutic efficiency (Sun et al., 2024).

To further validate the intestinal lymphatic transport mechanism of SME, rats were pretreated with CHX (3.0 mg/kg), an inhibitor that blocks chylomicron secretion in enterocytes, prior to the oral administration of either free DHA suspension or DHA-SME. As shown in Fig. 6D-F, CHX pretreatment significantly reduced the AUC_0__–__8_ (*P* < 0.001) and C_max_ (P < 0.001) of DHA after DHA-SME administration by 55.74% and 49.00%, respectively. In contrast, the pharmacokinetics of the DHA suspension remained virtually unchanged by CHX. These results suggested indirectly that SME relied primarily on the intestinal chylomicron assembly pathway for lymphatic transport, whereas free DHA was absorbed directly into the portal circulation. However, in view of the lack of direct lymphatic fluid sampling and quantification, this conclusion remains supportive rather than definitive.

### 3.14 In vivo antitumor efficacy against primary tumor and lung metatasis

**Fig. 7:**
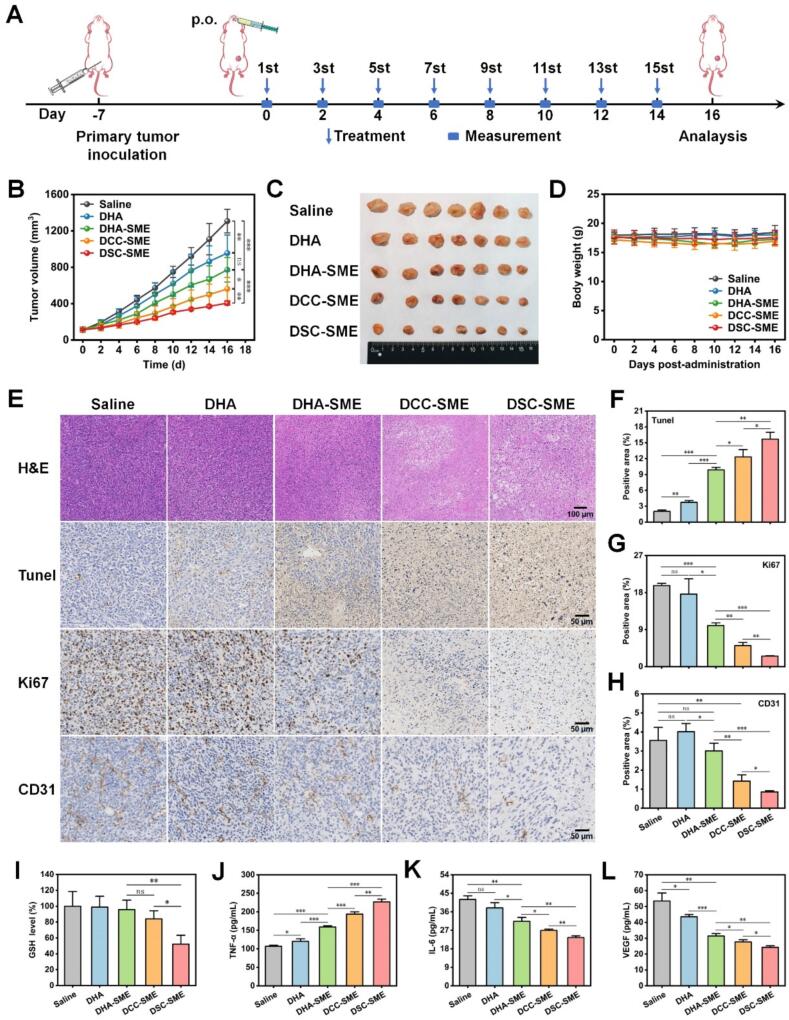
In vivo antitumor efficacy of DSC-SME against orthotopic 4 T1 tumor. (A) Schematic depiction of the experiment approach for the evaluation of the antitumor efficacy. (B) Tumor growth curves of 4 T1 tumor-bearing mice after treatment with DHA, DHA-SME, DCC-SME, or DSC-SME at the equivalent dose of 45 mg/kg DHA. Data are means ± SDs (*n* = 7). *P < 0.05, **P < 0.01, ***P < 0.001, ns, no significant difference. (C) Photographs of the excised tumors at the end of the treatment. (D) Body weights changes of the 4 T1 tumor-bearing mice during the treatment. Data are means ± SDs (n = 7). (E) Representative images of H&E staining, Tunel, Ki67 and CD31 immunohistochemical staining of tumor sections after treatment. Quantitative analysis of Tunel (F), Ki67 (G), and CD31 (H) positive areas of tumor sections after treatment. Data are means ± SDs (*n* = 3). *P < 0.05, **P < 0.01, ***P < 0.001, ns, no significant difference. (I) GSH levels in the excised tumors after treatment. Data are means ± SDs (n = 3). *P < 0.05, **P < 0.01, ns, no significant difference. Levels of TNF-α (J), IL-6 (K), and VEGF (L) in serum of 4 T1 tumor-bearing mice after treatment. Data are means ± SDs (n = 3). *P < 0.05, **P < 0.01, ***P < 0.001, ns, no significant difference.

The in vivo antitumor efficacies of drug-loaded SMEs were investigated in 4 T1 tumor-bearing mice at 45 mg/kg DHA for a total of fifteen doses (Fig. 7A). As shown in Fig. 7B-7D**, S12**, 4 T1 tumor-bearing mice in the saline group exhibited rapid tumor growth during the treatment. By 16th day, the tumor volume had reached approximately 1100 to 1500 mm^3^. Compared to the saline group, the other treatment groups demonstrated significant tumor growth inhibition (*P* < 0.01), with the extent of inhibition varying across groups. Among them, DSC-SME showed strongest tumor inhibitory effect in terms of tumor volume and tumor burden, which was consistent with the results of the in vitro cell apoptosis study and pharmacokinetic profile.

Furthermore, to explore the mechanisms of in vivo tumor growth inhibition elicited by drug-loaded SMEs, TUNEL Ki67, CD31, and H&E stainings were performed on the excised tumor tissue (Fig. 7E-H). In agreement with the in vitro cell apoptosis data (Fig. 4C), TUNEL staining for apoptotic cells demonstrated a marked increase in the number of apoptotic cells within the tumors following the treatment of DCC-SME or DSC-SME. Ki67 staining, a marker of cell proliferation, revealed pronounced positive staining (dark brown) in the saline control and free DHA groups, indicating highly active tumor cell proliferation. The DCC-SME or DSC-SME treatment both induced significantly lower levels of cell proliferation in comparison with DHA-SME (P < 0.01). Moreover, DCC-SME or DSC-SME treatment decreased the proportion of CD31-positive endothelial cells, implicating impaired angiogenesis as a potential factor in slowing tumor growth. H&E staining images showed an evident decrease in tumor cellular density following treatment with DCC-SME or DSC-SME, while a notable increase in necrotic area was observed. Critically, the disulfide bonds in DSC enabled a reduction-sensitive drug release specifically in the high-glutathione tumor microenvironment, while simultaneously lowering local GSH levels (Fig. 7I). And the deficiency of GSH can disrupt the redox homeostasis of cells, further leading to DHA induced ROS accumulation, ultimately causing tumor cell damage or even death (Liu et al., 2022). This combined effect underlay the enhanced anti-tumor performance of DSC-SME over DCC-SME. Taken together, the antitumor effects of DSC-SME are mediated through depletion of glutathione, promotion of apoptosis, inhibition of cell proliferation, and reduction of angiogenesis, which collectively contribute to its favorable therapeutic efficacy.

Inflammatory factors are pivotal in tumorigenesis and progression. Specifically, cytokines such as tumor necrosis factor-α (TNF-α) and interleukin-6 (IL-6) serve as key markers and mediators of the activated immune response within the tumor microenvironment (Cruceriu et al., 2020; Wu et al., 2025b). Concurrently, the high metabolic demand and inadequate blood supply of tumors create hypoxia. This hypoxic state upregulates pro-angiogenic factors like vascular endothelial growth factor (VEGF), which stimulates endothelial cell proliferation and promotes angiogenesis, thereby supporting further tumor growth (Apte et al., 2019). To this end, serum levels of TNF-α, IL-6, and VEGF in 4 T1 tumor-bearing mice from each treatment group were quantified using ELISA. As shown in Fig. 7J-L, treatment resulted in an upregulation of TNF-α alongside downregulations of IL-6 and VEGF. These cytokine shifts suggest that DHA and its prodrug-loaded SME not only enhanced tumor immunogenicity but also ameliorated the immunosuppressive tumor microenvironment (Li et al., 2024; Rao et al., 2024; Wu et al., 2019).

**Fig. 8:**
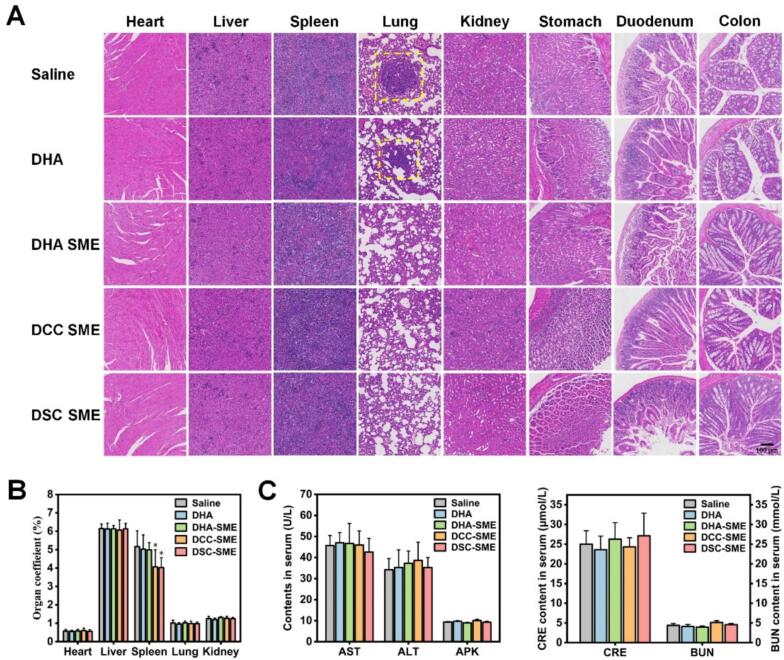
In vivo safety evaluation of DSC-SME. (A) Hematoxylin and eosin (H&E) staining images of tissue sections from heart, liver, spleen, lung, kidney, stomach, duodenum, and colon in 4 T1 tumor-bearing mice after treatment with DHA, DHA-SME, DCC-SME, or DSC-SME at the equivalent dose of 45 mg/kg DHA. (B) Organ coefficient of 4 T1 tumor-bearing mice after treatment. Data are means ± SDs (n = 7). *P < 0.05. (C) Levels of hepatic and renal function markers (AST, ALT, AKP, CRE, and BUN) in serum of 4 T1 tumor-bearing mice after treatment. Data are means ± SDs (*n* = 3).

While evaluating the antitumor efficacy of drug-loaded SMEs, we also conducted a preliminary safety assessment of their continuous oral administration. Safety was evaluated by monitoring changes in body weight, serum liver/kidney function biomarkers, and histopathological examination (H&E staining) of major organs from 4 T1 tumor-bearing mice. No mortality or adverse behavioral changes were observed in 4 T1 tumor-bearing mice during the 15-day treatment. Furthermore, body weights remained stable across all groups, with no significant inter-group differences. Upon completion of the dosing regimen, major organs (heart, liver, spleen, lungs, kidneys) were harvested, weighed, and the organ coefficients were calculated (Fig. 8A-B). Compared with the saline control, no significant differences were observed in the organ coefficients of the heart, liver, lungs, or kidneys across treatment groups. However, a significant reduction in spleen coefficient was noted in the prodrug-loaded SME groups (DCC-SME and DSC-SME). This decrease is often associated with reduced tumor burden and modulated systemic inflammation, indicating a favorable therapeutic outcome. Additionally, serum levels of hepatic and renal function markers (AST, ALT, AKP, CRE, and BUN) fell within the normal range and showed no significant differences among the treatment groups (Fig. 8C), indicating an absence of overt liver or kidney damage following administration. Importantly, H&E staining revealed the presence of metastatic lesions in the lungs of mice from both the saline control and DHA groups. In contrast, organs (heart, liver, spleen, lung, kidney, stomach, duodenum, and colon) from the SME-formulated treatment group appeared histologically normal, with no evident metastatic foci.

**Fig. 9:**
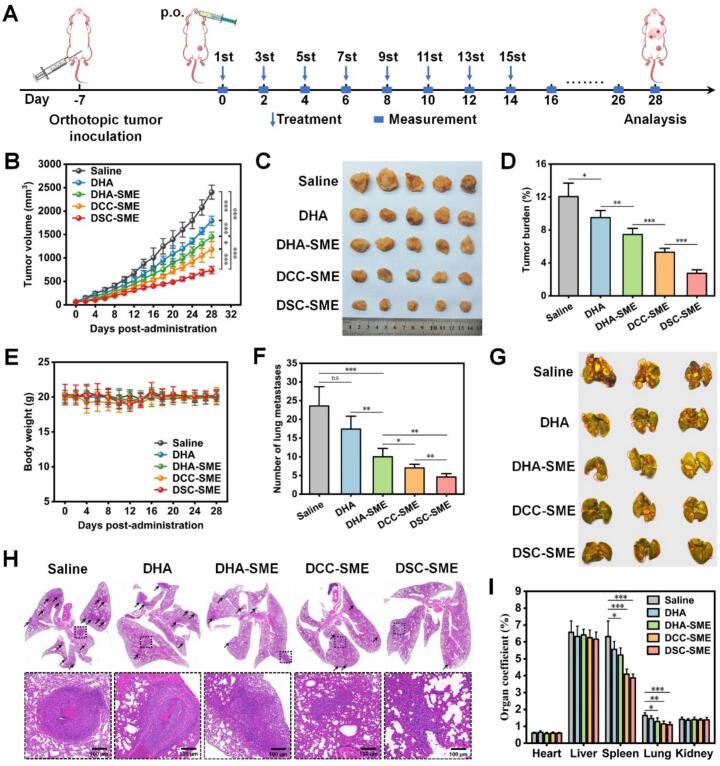
In vivo anti-metastatic efficacy of DSC-SME against orthotopic 4 T1 tumor. (A) Schematic depiction of the experiment approach to the lung metastases experiment. (B) Tumor growth curves of 4 T1 tumor-bearing mice after treatment with DHA, DHA-SME, DCC-SME, or DSC-SME at the equivalent dose of 45 mg/kg DHA. Data are means ± SDs (*n* = 5). *P < 0.05, ***P < 0.001. (C) Photographs of the excised tumors at the end of the treatment. (D) Tumor burden of 4 T1 tumor-bearing mice after treatment. Data are means ± SDs (n = 5). *P < 0.05, **P < 0.01, ***P < 0.001. (E) Body weights changes of the 4 T1 tumor-bearing mice during the treatment. Data are means ± SDs (n = 5). (F) Quantification of the lung metastasis from the treated 4 T1 tumor mice. (E) Representative images of H&E staining, Tunel, Ki67 and CD31 immunohistochemical staining of tumor sections after treatment. Quantitative analysis of Tunel (F), Ki67 (G), and CD31 (H) positive areas of tumor sections after treatment. Data are means ± SDs (n = 3). *P < 0.05, **P < 0.01, ***P < 0.001, ns, no significant difference. (I) GSH levels in the excised tumors after treatment. Data are means ± SDs (n = 3). *P < 0.05, **P < 0.01, ns, no significant difference. Levels of TNF-α (J), IL-6 (K), and VEGF (L) in serum of 4 T1 tumor-bearing mice after treatment. Data are means ± SDs (n = 3). *P < 0.05, **P < 0.01, ***P < 0.001, ns, no significant difference.

Given the presence of lung metastases, we further evaluated the therapeutic efficacy of prodrug-loaded SMEs against tumor metastatic. In this study, 4 T1 tumor-bearing mice received oral administrations of the prodrug-loaded SME (equivalent to 45 mg/kg DHA) once daily from day 0 to 15 (15 doses in total). Tumor volume and body weight were then monitored over a 28-day period, as outlined in the experimental timeline (Fig. 9A). DSC-SME inhibited tumor growth with the highest efficiency among the various treatments (Fig. 9B). At the end of study, the 4 T1 tumor-bearing mice were sacrificed to harvest the tumors for optical observation and weighting, and the smallest tumors were observed for the DSC-SME group (Fig. 9C-E). As shown in Fig. 9F-H, among all treatment, the DSC-SME group exhibited the fewest lung monastic nodules. This suggested that DSC-SME prevented the colonization of secondary sites (e.g., lungs) by disseminated tumor cells, underpinning its potent anti-metastatic efficacy (Iqbal et al., 2024; Tu et al., 2022). Additionally, no obvious change in the body weight was observed during the treatments, and the DSC-SME group showed significantly decreased spleen and lung coefficients relative to the saline control group (*P* < 0.001) (Fig. 9I). These data further support the dual benefits of safety and anti-metastasis effect for the prodrug-SME combination therapy. This superior efficacy is likely attributable to a synergistic enhancement of intestinal lymphatic drug transport. Upon entering the intestinal lymphatics, DSC-SME can target lymph-resident tumor cells or be delivered to tumor tissue via systemic lymphatic circulation. Subsequently, the high GSH levels in the tumor microenvironment trigger the reduction-sensitive release of active DHA, thereby exerting favorable anti-breast cancer efficacy and anti-metastatic effects.

## 4 Conclusion

In summary, we developed an SME formulation containing a disulfide bond-linked lipophilic DHA prodrug (DSC-SME) for oral delivery in breast cancer metastasis therapy. The DSC was designed with a disulfide bond as tumor microenvironment-sensitive linkage, which facilitated efficient loading into the SME formulation duo to its higher affinity and compatibility with the oil phase. The resulting DSC-SME possess appropriate droplet size, uniform distribution, high drug loading, good gastrointestinal and storage stability, and reduction-responsive drug release in vitro. Oral administered DSC-SME promoted intestinal lymphatic transport, likely via the chylomicron pathway, which may help bypass first-pass hepatic metabolism and improve oral bioavailability. In a murine orthotopic 4 T1 breast cancer model, DSC-SME showed antitumor efficacy against primary tumors and lung metastases, with no significant gastrointestinal or systemic toxicity observed under the tested conditions. Overall, these findings suggest that combining a disulfide-linked lipophilic DHA prodrug with an SME formulation represents a promising strategy for the development of oral anticancer formulations. Further studies are needed to evaluate its translational potential.

## CRediT authorship contribution statement

**Bin Zheng:** Writing – review & editing, Writing – original draft, Project administration, Funding acquisition, Conceptualization. **Cuiping He:** Visualization, Methodology, Formal analysis. **Fengye Zhao:** Visualization, Validation. **Ran Li:** Validation, Investigation. **Ziyi Zhang:** Validation, Formal analysis. **Xiaojie Chen:** Methodology, Investigation. **Minfei Shi:** Software, Methodology. **Beibei He:** Methodology, Formal analysis. **Rongrong Wang:** Resources, Data curation. **Guolian Ren:** Supervision, Resources, Project administration. **Shuqiu Zhang:** Writing – review & editing, Supervision, Resources, Project administration. **Shuang Yang:** Writing – review & editing, Supervision, Project administration, Funding acquisition, Data curation, Conceptualization.

## Declaration of competing interest

The authors declare the following financial interests/personal relationships which may be considered as potential competing interests:

Bin Zheng reports financial support was provided by Shanxi Provincial Department of Science and Technology. Shuang Yang reports financial support was provided by Shanxi Provincial Department of Science and Technology. Guolian Ren reports financial support was provided by Shanxi Provincial Department of Science and Technology. If there are other authors, they declare that they have no known competing financial interests or personal relationships that could have appeared to influence the work reported in this paper.

## Data Availability

Data will be made available on request.
