# Supplementary material for: A reduction-sensitive lipophilic dihydroartemisinin prodrug in a self-microemulsifying drug delivery system for treating breast cancer lung metastasis via intestinal lymphatic transport
Source: Int J Pharm X. 2026 May 3;11:100556. doi: 10.1016/j.ijpx.2026.100556 (PMC13156721; doi:10.1016/j.ijpx.2026.100556)
Supplement: Supplementary file 1 — Supplementary material [file mmc1.docx]

Supplementary Material

**A Reduction-Sensitive Lipophilic** **Dihydroartemisinin Prodrug in a Self-Microemulsifying Drug Delivery System for Treating Breast Cancer Lung Metastasis via Intestinal Lymphatic Transport**

Bin Zheng^1,^^2^^†^, Cuiping He^1,2†^, Fengye Zhao^1,2^, Ran Li^1,2^, Ziyi Zhang^1,2^, Xiaojie Chen^1,2^, Minfei Shi^1,2^, Beibei He^1,2^, Rongrong Wang^1,2^, Guolian Ren^1,2^*, Shuqiu Zhang^1,2^*, Shuang Yang^1,3^*

^1^Medicinal Basic Research Innovation Center of Chronic Kidney Disease, Ministry of Education, School of Pharmacy, Shanxi Medical University, Taiyuan 030001, China

^2^Shanxi Provincial Key Laboratory of Drug Synthesis and Novel Pharmaceutical Preparation Technology, School of Pharmacy, Shanxi Medical University, Taiyuan 030001, China

^3^School of Basic Medical Sciences, Shanxi Medical University, Taiyuan 030001, China

^†^Bin Zheng and Cuiping He contributed equally to this work.

*Correspondence:

Shuang Yang, [yangshuangzz@126.com](mailto:yangshuangzz@126.com);

Shuqiu Zhang, [shuqiu.zhang@sxmu.edu.cn](mailto:shuqiu.zhang@sxmu.edu.cn).

Guolian Ren, glren2007@126.com

**Figure S1** Synthetic routes of DHA-CC-C18 (DCC)

**Figure S2** Synthetic routes of DHA-SS-C18 (DSC)


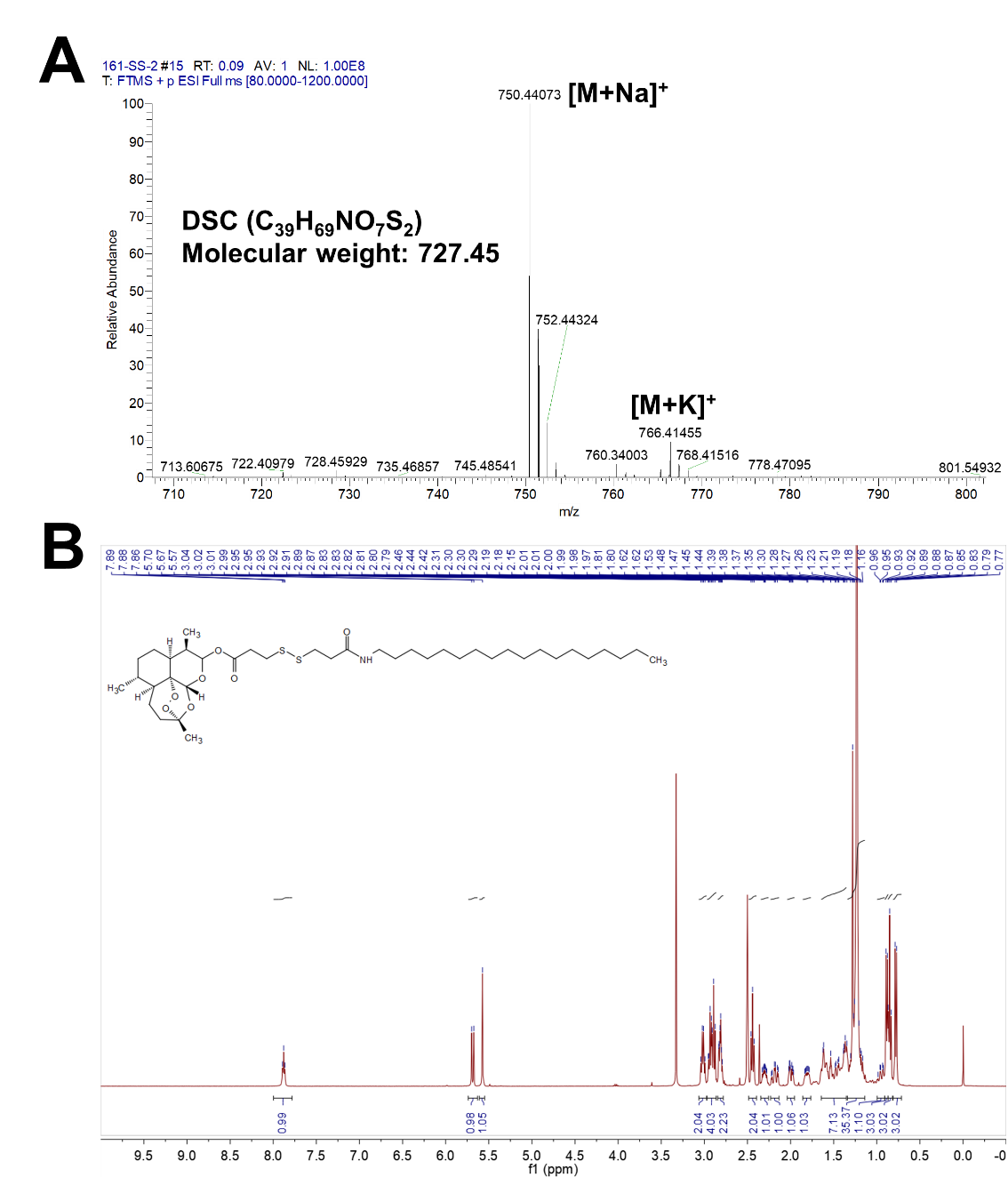


**Figure S3** (A) ESI-HRMS and (B) ^1^H NMR (400 MHz, DMSO-*d_6_*) of DSC.

HRMS (ESI) *m/z* for C_39_H_69_NO_7_S_2_Na [M+Na]^+^ : 750.44073.

^1^H NMR (400 MHz, DMSO-*d*_6_) δ 7.88 (t, *J* = 5.6 Hz, 1H), 5.69 (d, *J* = 9.8 Hz, 1H), 5.57 (s, 1H), 3.02 (q, *J* = 6.9 Hz, 2H), 2.96 – 2.86 (m, 4H), 2.85 – 2.78 (m, 2H), 2.44 (t, *J* = 7.0 Hz, 2H), 2.34 – 2.26 (m, 1H), 2.18 (td, *J* = 13.9, 13.4, 4.0 Hz, 1H), 2.04 – 1.96 (m, 1H), 1.86 – 1.76 (m, 1H), 1.64 – 1.35 (m, 7H), 1.34 – 1.13 (m, 35H), 1.00 – 0.91 (m, 1H), 0.88 (d, *J* = 6.5 Hz, 3H), 0.85 (t, *J* = 7.2 Hz,, 3H), 0.78 (d, *J* = 7.2 Hz, 3H).


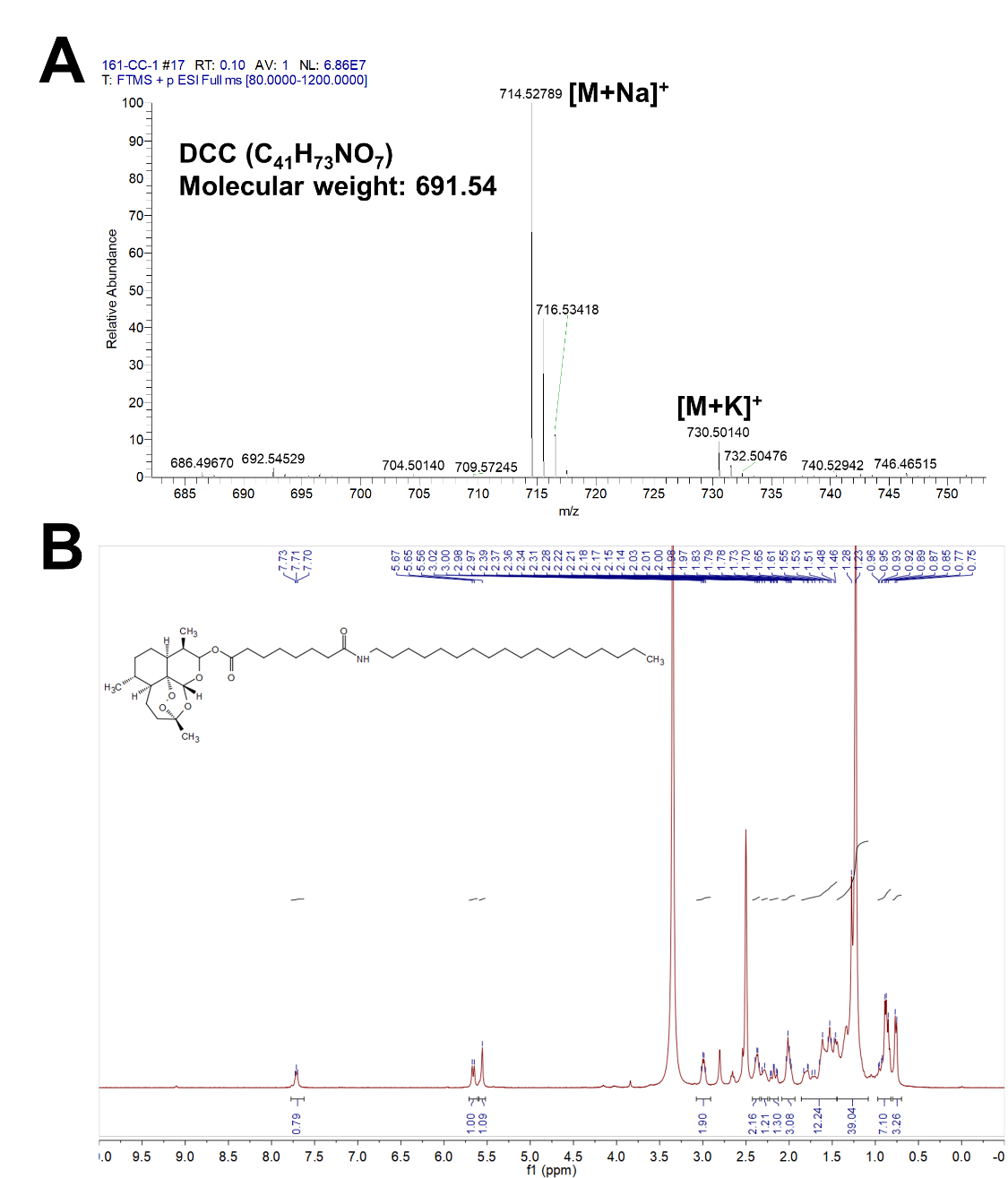


**Figure S4** (A) ESI-HRMS and (B) ^1^H NMR (400 MHz, DMSO-*d_6_*) of DCC.

HRMS (ESI) *m/z* for C_41_H_73_NO_7_Na [M+Na]^+^ : 714.52789.

^1^H NMR (400 MHz, DMSO-*d*_6_) δ 7.71 (t, J = 5.8 Hz, 1H), 5.66 (d, J = 9.7 Hz, 1H), 5.56 (s, 1H), 2.99 (q, J = 6.7 Hz, 2H), 2.41 – 2.33 (m, 2H), 2.32 – 2.26 (m, 1H), 2.21 – 2.16 (m, 1H), 2.09 – 1.94 (m, 3H), 1.86 – 1.45 (m, 12H), 1.45 – 1.08 (m, 39H), 0.97 – 0.82 (m, 7H), 0.76 (d, J = 7.1 Hz, 3H).


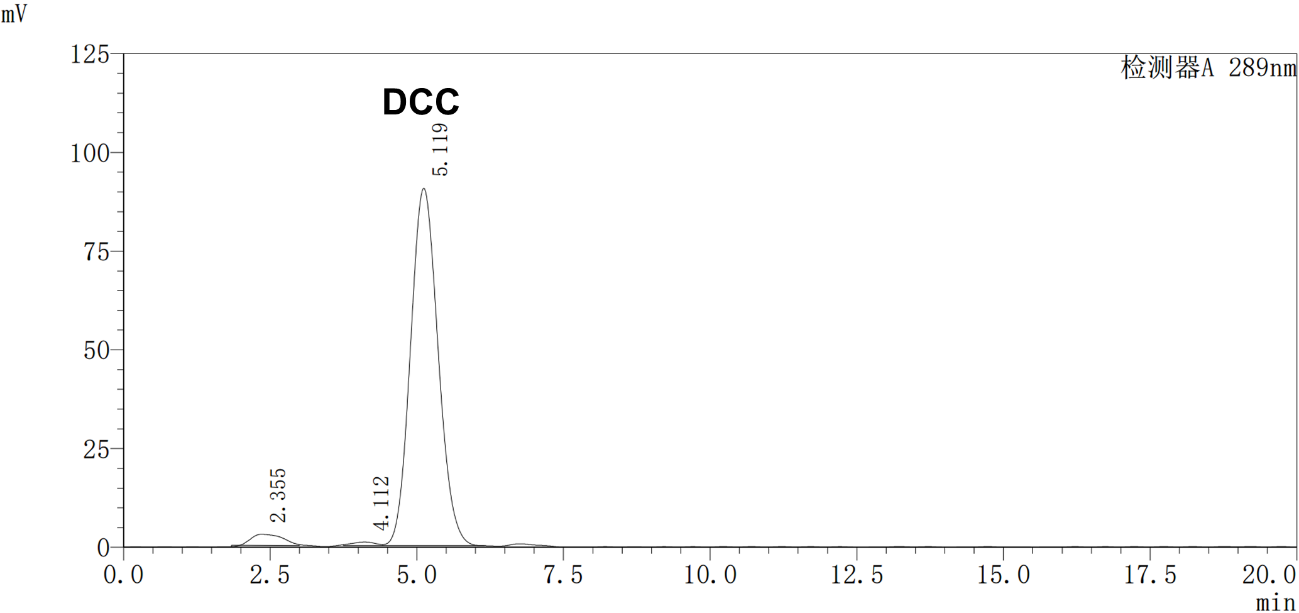


**Figure S5** HPLC chromatogram of DCC.

The purity was determined to be 95.88% based on peak area normalization (retention time: approximately 5.119 min).


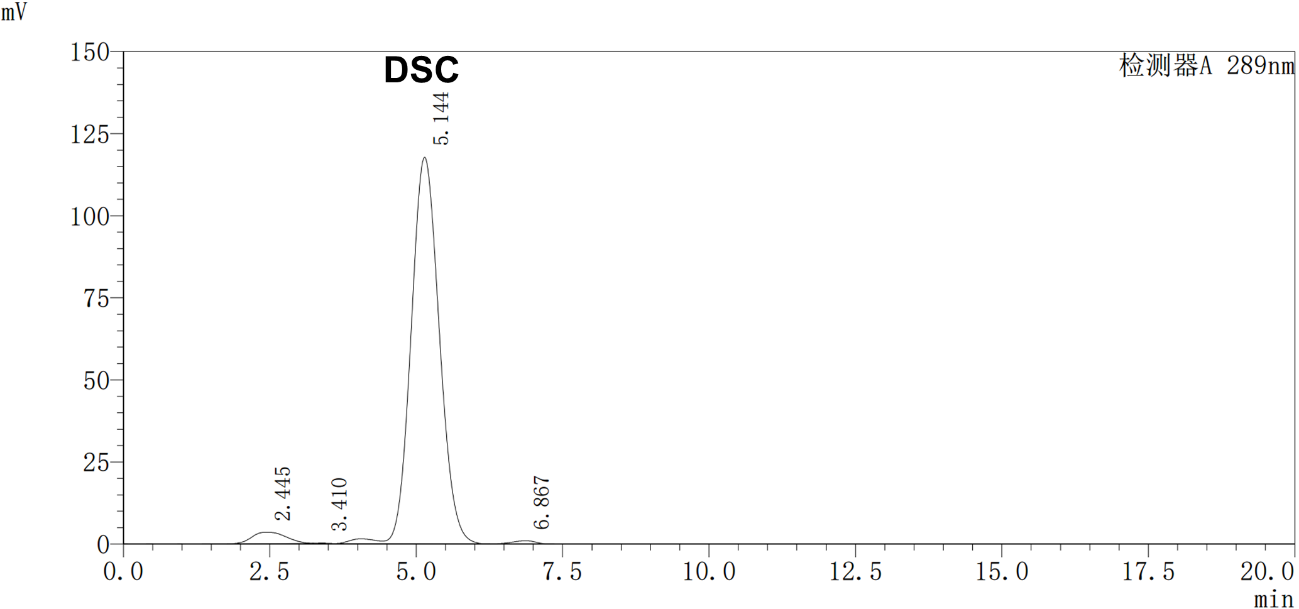


**Figure S6** HPLC chromatogram of DSC.

The purity was determined to be 95.68% based on peak area normalization (retention time: approximately 5.144 min).


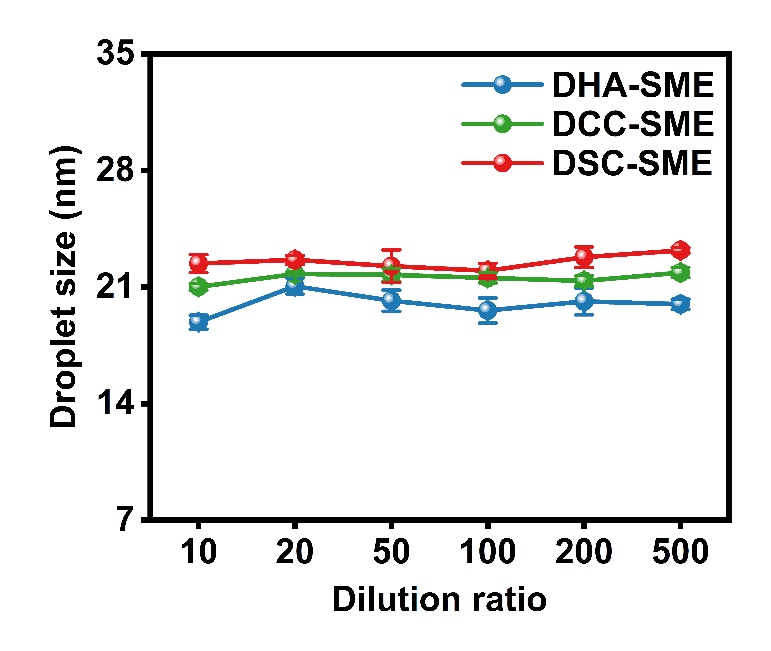


**Figure S7** Dilution stability of DHA-SME, DCC-SME, and DSC-SME in diluted hydrochloric acid (pH 1.2). Data are means ± SDs (n=3).


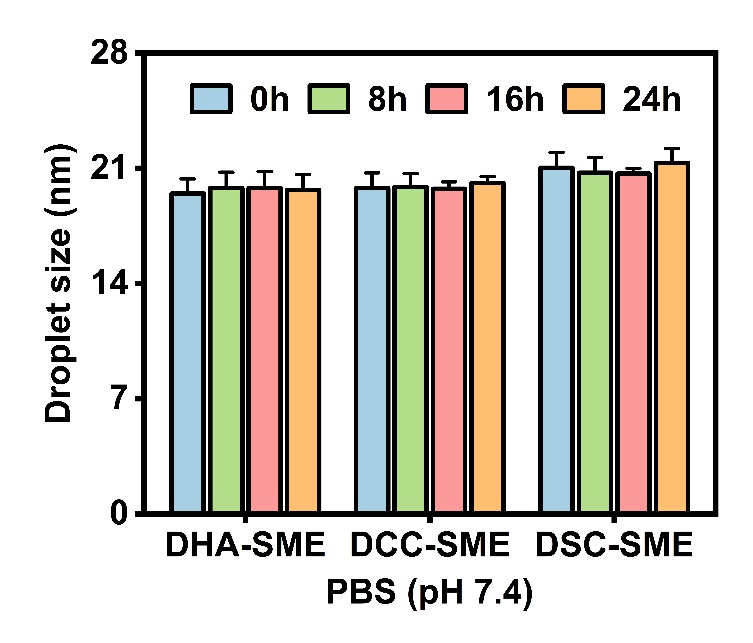


**Figure S8** Medium stability of DHA-SME, DCC-SME, and DSC-SME after incubation in phosphate buffer saline (PBS, pH 7.4). Data are means ± SDs (n=3).


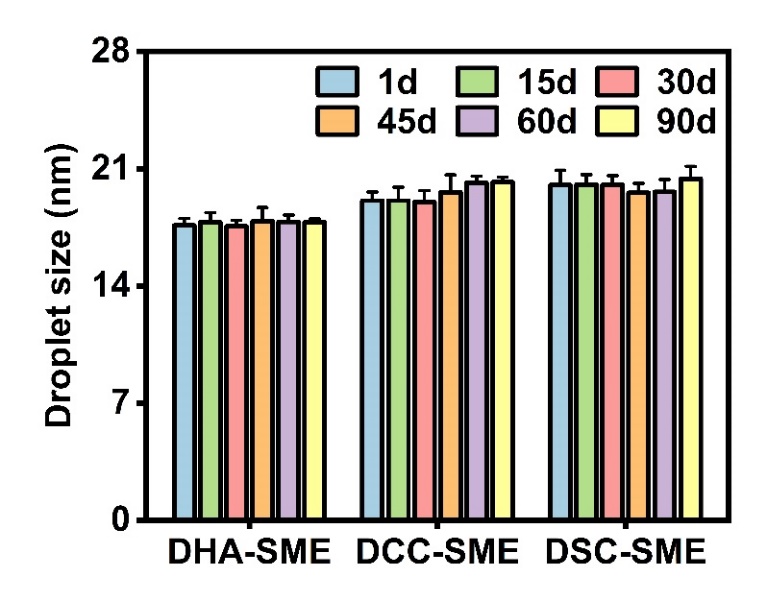


**Figure S9** Storage stability of DHA-SME, DCC-SME, and DSC-SME after 90 days of storage at room temperature. Data are means ± SDs (n=3).


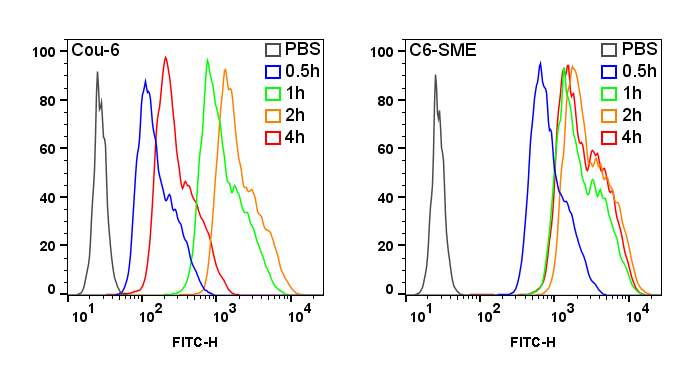


**Figure S10** Flow cytometric profiles of Cou-6 and C6-SME in Caco-2 cells after incubation for various time intervals at 37 °C.


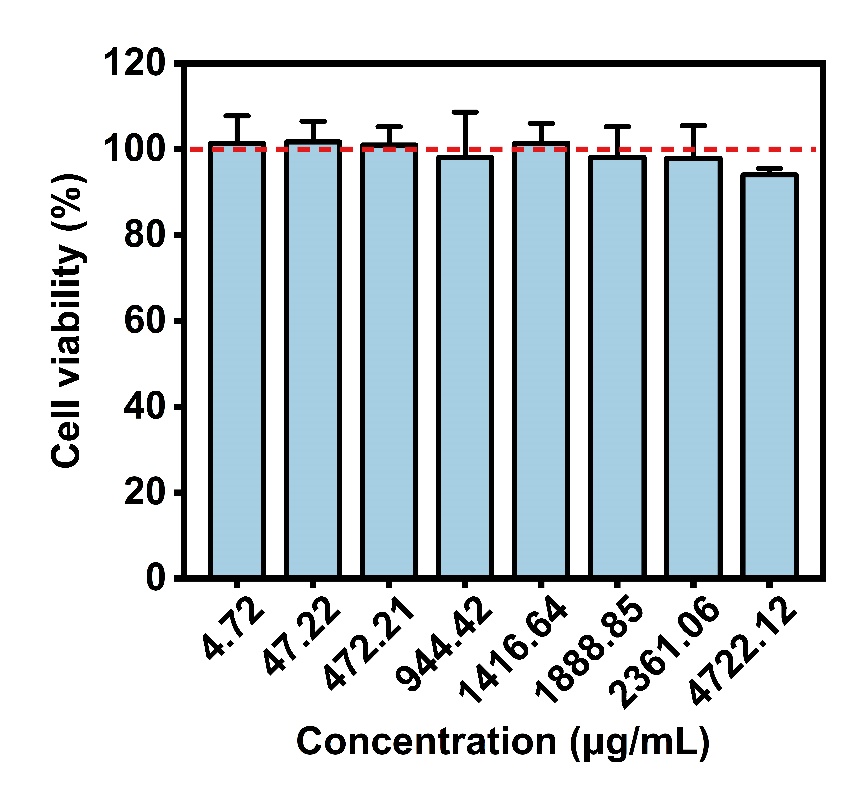


**Figure S11** Cell viability of Caco-2 cells after treatment with various concentration of SME for 24 h. Data are means ± SDs (n=5).


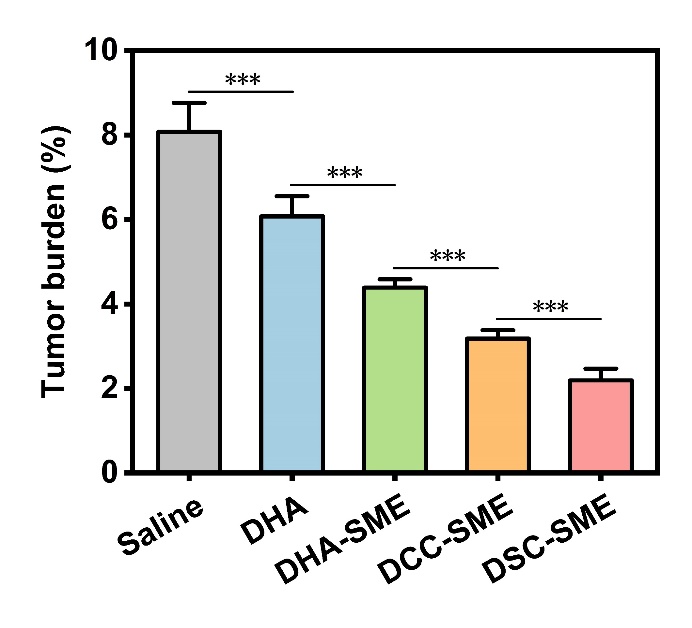


**Figure S12** Tumor burden of 4T1 tumor-bearing mice after treatment with DHA, DHA-SME, DCC-SME, or DSC-SME at the equivalent dose of 45 mg/kg DHA. Data are means ± SDs (n=7). ***P < 0.001.

**Table S1** Compatibility and grade results of oil phase MCT and surfactants

| Surfactants | Emulsification levels in various proportions (MCT:surfactant, w/w) | | | | |
| --- | --- | --- | --- | --- | --- |
|  | 1:9 | 2:8 | 3:7 | 4:6 | 5:5 |
| Tween-20 | I | III | III | IV | IV |
| Tween-80 | I | II | III | IV | V |
| OP-10 | I | II | III | IV | V |
| Cremophor EL | I | I | I | II | III |
| RH40 | I | I | I | II | IV |
| Labrasol | I | IIII | IV | V | V |

**Table** **S2** Compatibility results of surfactants and cosurfactants

| Cosurfactants | Surfactants | | | | | |
| --- | --- | --- | --- | --- | --- | --- |
|  | Tween-20 | Tween-80 | OP-10 | Cremophor EL | RH40 | Labrasol |
| Transcutol HP | P | P | P | P | P | P |
| n-Butanol | P | P | P | P | P | P |

**Table S3** Characterization of drug-loaded SMEs and their microemulsions (n=3)

| Formulation | DHA-SME | DCC-SME | DSC-SME |
| --- | --- | --- | --- |
| Droplet size (nm) | 17.72 ± 0.19 | 18.60 ± 0.13 | 20.03 ± 0.54 |
| PDI | 0.05 ± 0.01 | 0.03 ± 0.02 | 0.04 ± 0.01 |
| Zeta potential (mV) | -0.85 ± 0.03 | -1.92 ± 0.10 | -2.38 ± 0.29 |
| Equilibrium solubility (mg/g) | 20.04 ± 1.24 | 47.22 ± 2.03 | 84.82 ± 2.69 |
| Drug loading capacity (mg/g) | 12.00 | 34.00 | 56.00 |
| Drug loading capacity (mmol/g) | 0.042 | 0.049 | 0.077 |
| Encapsulation efficiency (%) | 99.48 ± 1.25 | 99.69 ± 1.99 | 99.36 ± 1.58 |

**Table S4**  IC_50_ values (nmol/mL) of DHA, DCC, DSC and corresponding drug-loaded SMEs against three tumor cell lines (n=5)

| Cell lines | DHA | DHA-SME | DCC | DCC-SME | DSC | DSC-SME |
| --- | --- | --- | --- | --- | --- | --- |
| 4T1 | 42.94 ± 2.40 | 30.85 ± 1.29 | 89.49 ± 8.39 | 44.43 ± 3.92 | 73.89 ± 4.96 | 36.53 ± 2.06 |
| MCF-7 | 41.39 ± 2.20 | 34.00 ± 1.31 | 101.02 ± 10.63 | 75.06 ± 6.60 | 73.73 ± 3.67 | 60.06 ± 2.07 |
| HepG2 | 32.50 ± 3.18 | 21.59 ± 4.04 | 86.13 ± 13.11 | 66.16 ± 8.67 | 68.84 ± 4.53 | 48.00 ± 4.20 |

**Table S5**  Pharmacokinetic parameters of DHA, DHA-SME, DCC-SME and DSC-SME (n=5)

| Formulations | Determined | C_max_ (nmol/L) | T_max_ (h) | AUC_0-24_ (nmol/L*h) | MRT_0-24_ (h) |
| --- | --- | --- | --- | --- | --- |
| DHA | DHA | 1307.27±129.73 | 0.65±0.14 | 1898.69±127.09 | 2.92±0.36 |
| DHA-SME | DHA | 2066.26±244.61 | 1.60±0.55 | 7909.65±1793.26 | 3.85±0.23 |
| DCC-SME | DHA | 361.59±39.47 | 4.40±0.89 | 2185.87±360.27 | 4.85±0.29 |
|  | DCC | 2155.03±241.50 | 2.80±1.10 | 10814.55±2182.68 | 4.97±0.24 |
| DSC-SME | DHA | 808.43±145.29 | 4.80±1.10 | 4998.51±889.87 | 5.18±0.36 |
|  | DSC | 1727.54±336.87 | 3.60±0.89 | 11918.18±1967.55 | 6.18±0.43 |
